# High-sensitive and disposable myocardial infarction biomarker immunosensor with optofluidic microtubule lasing

**DOI:** 10.1515/nanoph-2022-0208

**Published:** 2022-06-07

**Authors:** Panpan Niu, Junfeng Jiang, Kun Liu, Shuang Wang, Tong Wang, Yize Liu, Xuezhi Zhang, Zhenyang Ding, Tiegen Liu

**Affiliations:** School of Precision Instrument and Opto-electronics Engineering, Tianjin University, Tianjin 300072, China; Key Laboratory of Opto-electronics Information Technology (Tianjin University), Key Laboratory of Micro Opto-electro Mechanical System Technology (Tianjin University), Ministry of Education, Tianjin 300072, China; Tianjin Optical Fiber Sensing Engineering Center, Institute of Optical Fiber Sensing of Tianjin University, Tianjin 300072, China

**Keywords:** biomarker, cardiac troponin, immunosensor, optofluidic, whispering gallery mode

## Abstract

The early diagnosis of myocardial infarction can significantly improve the survival rate in emergency treatment, which is mainly implemented by the immunoassay for myocardial infarction biomarkers such as cardiac troponins in blood. In this work, a disposable optofluidic microtubule whispering gallery mode (WGM) immunosensor for label-free cardiac troponin I-C (cTnI-C) complex detection has been proposed and demonstrated with active interrogation enhancement. The disposable microtubule is simply fabricated by a silica capillary with pressurized tapering technology for thin-wall, and the cTnI antibodies are immobilized on the inner wall surface of the microtubule through the self-adherent polydopamine substrate. By configuring the two coupling microfibers, the double-fiber-coupled microtubule cavity can serve as a tunable filter for the mutual-coupled polarimetric fiber ring laser (FRL), whose output laser wavelength is determined by the cTnI-C concentration in the optofluidic microtubule with inherent microfluidic channel. Due to the cyclic-cumulative gain of the FRL, the characteristic resonant peak of optical sensing signal is enhanced in the spectral width compression and the optical signal-to-noise ratio improvement, and therefore the optical immunosensor for cTnI-C can be achieved by tracking the output laser wavelength of the FRL conveniently. The dynamic binding and unbinding process of cTnI-C antigen–antibody is illustrated by monitoring the lasing peak wavelength continuously. Our all-fiber immunosensor demonstrated here has the advantages of fast label-free detection, real-time monitor, high sensitivity and disposable sensing element, which can be an innovative detecting tool in early diagnosis of myocardial infarction.

## 1 Introduction

According to a statistic from the American Heart Association, the mortality rate of myocardial infarction (MI) is as high as ∼14% for people who experience an MI [1]. MI is a cardiovascular disease characterized by the death of cardiomyocytes, which is mainly caused by the imbalance between the demand for blood by the cardiac tissue and the supply of blood [2]. American Heart Association and American College of Cardiology recommend the administration of therapy to eligible patients within 12 h of the symptom onset [3]. Therefore, timely detection of blood-based cardiac biomarker based on the current MI care standard [3], is important for deciding the need for hospitalization and assisting in the selection of an optimal treatment for patients presenting with symptom onset. The common MI biomarkers include cardiac troponin I (cTnI), creatine kinase, lactate dehydrogenase and myoglobin [4]. Among those biomarkers for MI, cTnI not only has the high specificity of cardiomyocyte but also provide a long-lasting detection window for myocardial damage, which has been recognized as the principal biomarker for MI diagnosis [5, 6]. In the MI patient blood, cTnI is found in complex with troponin C as the cardiac troponin I-C (cTnI-C) [7–9], and the cTnI-C complex is preferable for the protein standards and calibrators owing the high stability and dominance in blood [10, 11]. The early diagnosis of MI through measuring the cTnI-C complex in blood, can promote life-saving and life-extending interventions, where rapid treatment usually requires a less invasive intervention procedure [3, 12, 13].

Nowadays, various analytical methods have been developed for quantification detection of cTnI, including enzyme-linked immunosorbent assays [14, 15] as well as chemiluminescence [16, 17], fluorescence [18, 19], electrochemiluminescence [20, 21], colorimetric [22, 23], surface plasmon resonance (SPR) [24, 25], and optical fiber-based immunosensors [26–29]. Compared to other methods, optical fiber-based immunosensors have inherent advantages of fast response, miniature size, easy fabrication, anti-electromagnetic interference and electrical passive. For optical fiber-based immunosensors, the optical fibers which are mostly made of biocompatible silica and plastic materials, serve both as the optical waveguides and the sensing elements, hence the diversity of sensing mechanisms could be realized by modifying the fiber structure with different configurations [30]. Masson et al. reported a multi-mode fiber SPR probe to detect cTnI [26], the SPR on the deposited gold film is excited to monitor biomolecular interactions between the cTnI antibodies and antigens, and a detection limit of 1.4 ng/mL was obtained. Zhou et al. used the optical microfiber coupler to build a cTnI biosensor with the interference turning effect [27], a high sensitivity within a small detection range of 2–10 fg/mL was achieved by operating the sensor works at the turning point. Liu et al. reported fiber-optic evanescent field biosensor based on phase-shifted microfiber Bragg grating [28], the fine reflective spectrum induced by the phase shift in modulation could improve the spectral resolution for cTnI concentration measurement. Ran et al. exploited a harmonic grating immunosensor for cTnI detection [29], the harmonic resonances of the microfiber grating with different responses could reduce the impact of the thermal noise. However, the wavelength-modulated immunosensors based on optical fibers are mostly implemented by tracking the characteristic resonant wavelengths in the transmission/reflection spectra [31–33], and the measuring performances of immunosensors with passive sensing configuration are restricted to the flat sensing spectra with broad width [34–36], especially for the slight wavelength changes caused by the biomarker concentration variations, in the common wavelength demodulation. The recognizable single-wavelength laser with narrow full width at half maximum (FWHM) and high optical signal-to-noise ratio (OSNR) [37–39], is instrumental in discriminating the wavelength shifts for high-resolution cTnI detection. Moreover, additional microfluidic channel packages are required for the fiber-optic cTnI biosensors mentioned above to compatible with the popular high-throughput microfluidic test scheme [40, 41].

In recent years, whispering gallery mode (WGM) microcavities based on different resonator structures have attracted wide attention in high-sensitivity biosensing due to their unique properties of small mode volume and high quality factor [42–44]. However, WGM resonators with high *Q* factors need rigorous manufacturing processes, and the sensing spectra of WGM immunosensors may degrade after functionalization. Herein, we developed a fiber-optic cTnI-C immunosensor based on an optofluidic microtubule WGM cavity with active interrogation enhancement. With polydopamine (PDA)-assisted immobilization, the disposable optofluidic microcavity which is fabricated with pressurized tapering technology was employed as the sensing element and the microfluidic channel simultaneously. By the combination of the mutual-coupled polarimetric fiber ring laser (FRL), the double-fiber-coupled microcavity as an optical filter to tune the output wavelength from FRL, and the generated sensing laser with narrow spectral width and high optical power is directly related to the cTnI-C concentration in the disposable sample microtubule. Analytical performance of the present immunosensor was evaluated by recording the spectrum evolution of the sensing laser, and the real-time monitor of the dynamic binding and unbinding process of cTnI-C antigen-antibody is achieved by measuring the lasing peak wavelength continuously. The fiber-optic immunosensor demonstrated here provides a proof-of-concept to continuous biomarker detection enabling companion diagnosis with emergency treatments for MI at the incipient stage.

## 2 Materials and methods

### 2.1 Materials and reagents

Fused silica capillary (Catalog No. TSP250350) was fabricated by Polymicro Technologies (Phoenix, USA). Standard communication single-mode fiber (SMF, Catalog No. SMF-28e) was fabricated by Corning Inc. (New York, USA). Tris (hydroxymethyl) aminomethane (Catalog No. T8060), dopamine hydrochloride (Catalog No. D9520), bovine serum albumin (BSA, Catalog No. A8020), goat anti-rabbit IgG (Catalog No. SPA134), recombinant human C-reactive protein (CRP) were purchased from Solarbio Science and Technology Co., Ltd. (Beijing, China). Protein elution buffer (0.1 M pH3.5 glycine-HCl buffer, Catalog No. MP005M) was purchased from M&C Gene Technology LTD. (Beijing, China). Deionized water (Catalog No. W820537) was purchased from Macklin Biochemical Co., Ltd. (Shanghai, China). Human prostate specific antigen (PSA, Catalog No. CSB-DP274I) was purchased from Huamei Biotech Co., Ltd. (Wuhan, China). FITC-conjugated monoclonal mouse anti-cardiac troponin I antibody (Catalog No. V3201-FITC), recombinant human cardiac troponin I-C (cTnI-C) complex protein (Catalog No. bs-41212P) and phosphate-buffered saline (PBS) solution (0.01 M pH 7.2–7.4, Catalog No. C01-01001) were purchased from Biosynthesis Biotechnology Inc. (Beijing, China). The liquid cTnI-C samples were prepared by diluting the cTnI-C complex protein with PBS solution in the concentration range of 0.05–0.55 ng/mL with an interval of 0.05 ng/mL.

### 2.2 Principle and fabrication of the double-fiber-coupled WGM microtubule cavity

**Figure 1:**
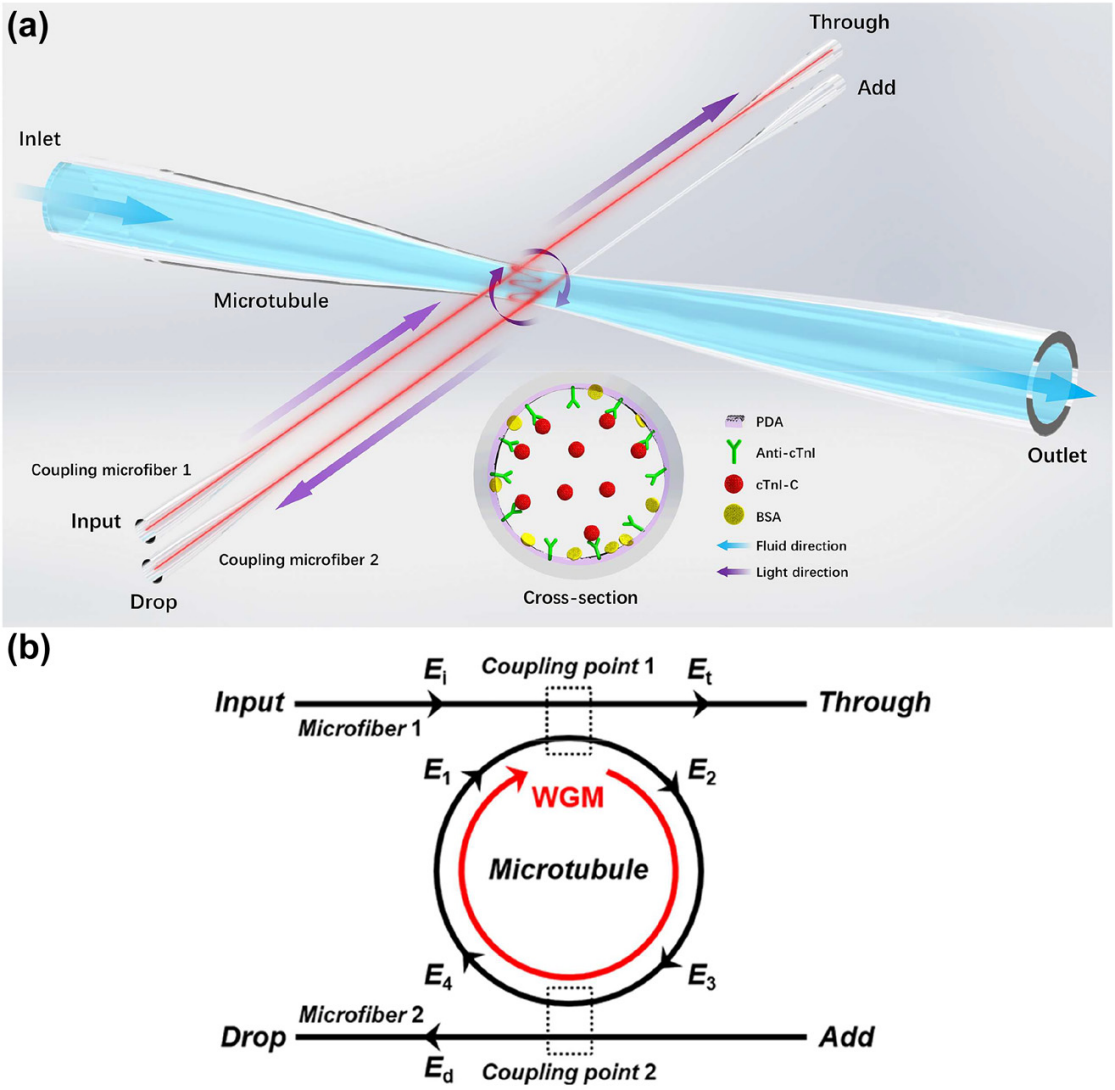
Double-fiber-coupled microtubule immunosensor. (a) Schematic illustration of the disposable immunosensor based on double-fiber-coupled WGM microtubule cavity. Inset: cross-section of the microtubule cavity with PDA functionalization. Inset: cross-section diagram of the functionalized microtubule. (b) Optical filter model for the double-fiber-coupled microtubule cavity.

The proposed disposable immunosensor based on double-fiber-coupled WGM microtubule cavity consists of a functionalized thin-wall microtubule and two coupling microfibers, as shown in Figure 1(a). The two coupling microfibers were orthogonally arrayed with the axial direction of the microtubule, and the cross-sectional ring waveguide of the microtubule with the symmetrical coupling paths could be considered as an optical filter as show in Figure 1(b). The light fields at different positions of the filter are *E*_*p*_ (*p* = 1, 2, 3, 4). Part of the *E*_i_ from the input port enters the microtubule at the coupling point 1 of the microfiber 1, the *E*_t_ output from through port and the *E*_2_ entering the microtubule can be expressed as follow:

(1) $$\left[\begin{array}{c} E_{\text{t}}\\ E_{2} \end{array}\right]=\left[\begin{array}{cc} t_{1} & k_{1}\\ -k_{1}^{*} & t_{1}^{*} \end{array}\right]\left[\begin{array}{c} E_{\text{i}}\\ E_{1} \end{array}\right]$$

(2) $$\left[\begin{array}{c} E_{\text{d}}\\ E_{4} \end{array}\right]=\left[\begin{array}{cc} t_{2} & k_{2}\\ -k_{2}^{*} & t_{2}^{*} \end{array}\right]\left[\begin{array}{c} E_{\text{a}}\\ E_{3} \end{array}\right]$$

where *t*_*i*_ (*i* = 1, 2) and *k*_*i*_ (*i* = 1, 2) are the transmission coefficient and coupling coefficient of coupling points, and satisfied ${\left|t_{i}\right|}^{2}+{\left|k_{i}\right|}^{2}=1,\left(i=1,2\right)$ , ^*^ represents conjugation. The normalized output light intensities from the through port *P*_t_ and the drop port *P*_d_ are:

(3) $$P_{\text{t}}={\left|E_{\text{t}}\right|}^{2}={\left|\frac{t_{1}-t_{2}\alpha {\text{e}}^{\text{i}\theta }}{1-t_{1}t_{2}\alpha {\text{e}}^{\text{i}\theta }}\right|}^{2}=\frac{t_{1}^{2}-2\alpha t_{1}t_{2}\;\cos\;\theta +\alpha^{2}t_{2}^{2}}{1-2\alpha t_{1}t_{2}\;\cos\;\theta +\alpha^{2}t_{1}t_{2}^{2}}$$

(4) $$\begin{array}{ll} P_{\text{d}} & ={\left|E_{\text{d}}\right|}^{2}={\left|\frac{-k_{1}k_{2}\sqrt{\alpha }e^{\text{i}\theta /2}}{1-t_{1}t_{2}\alpha {\text{e}}^{\text{i}\theta }}\right|}^{2}=\frac{\alpha k_{1}^{2}k_{2}^{2}}{1-2\alpha t_{1}t_{2}\;\cos\;\theta +\alpha^{2}t_{1}t_{2}^{2}} \end{array}$$

where *θ* = 2π*kRn*_eff_, *k* is the wave vector, *n*_eff_ and *R* are the effective refractive index and the equivalent radius of the ring waveguide, respectively.

**Figure 2:**
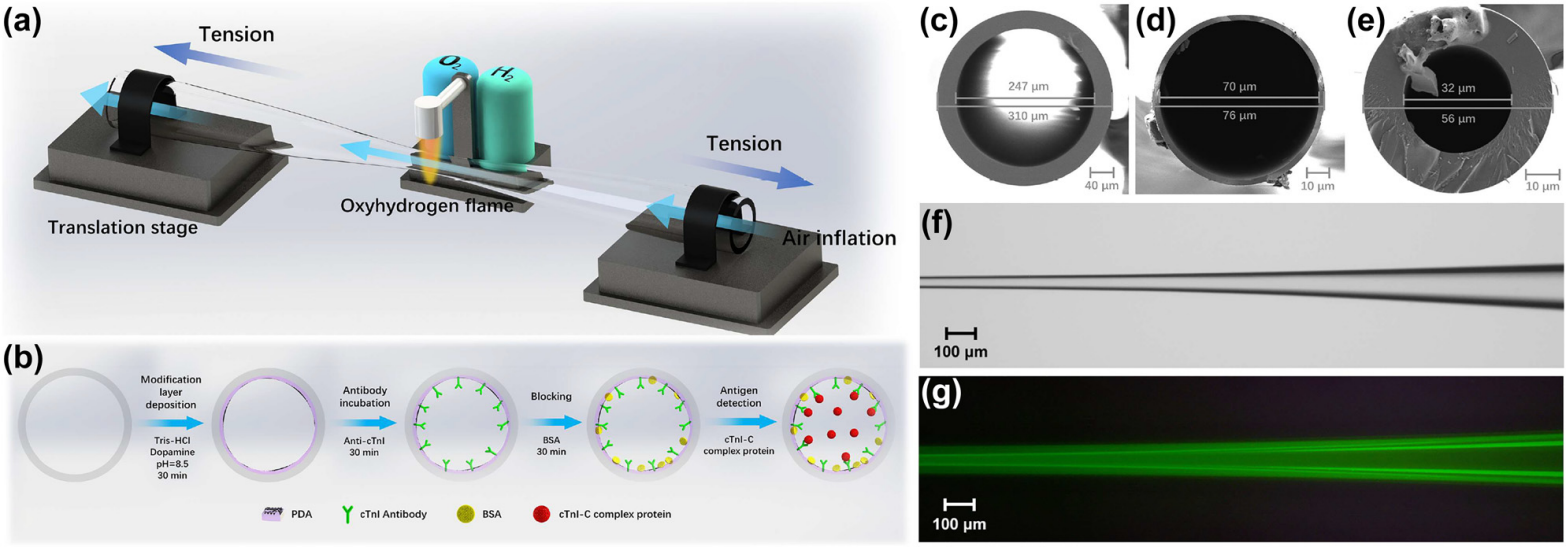
Schematic diagrams of (a) the microtubule tapering with pressurization and (b) the functionalization for the microtubule with PDA. SEM images of (c) the untapered capillary, (d) the tapered capillary with pressurization and (e) the tapered capillary without pressurization. (f) Optical and (g) fluorescence microscopic images of the microtubule immobilized FITC-conjugated cTnI antibody.

The disposable microtubule and the microfibers were tapered by using the fused silica capillary and the single-mode fiber with oxyhydrogen flame, respectively. The diameters of microfibers with losses less than 0.2 dB were subwavelength ∼1 μm for high coupling efficiency. During the tapering process of microtubule, the pressure inside the microtubule is controlled by a pneumatic pump for a thin-wall waist as shown in Figure 2(a), which induces more evanescent fields to penetrate into the surrounding [45], thereby enhancing the detecting sensitivity to the medium in microtubule. In order to realize the specific capture of the targeted cTnI-C, a modified layer based on PDA, as shown in the inset of Figure 1(a), was built on the inner wall surface of the prepared microtubule, and the functionalization of the microtubule followed the steps below (as shown in Figure 2(b)).

(1) Deposition of PDA substrate. The microtubule was filled with 3 mg/L tris-buffered dopamine hydrochloride solution for 30 min standing.
(2) Decoration of the immunological recognition. 10 μg/mL anti-cTnI PBS solution was injected into the microtubule for 30 min incubation.
(3) Blocking the nonspecific sites. 0.1 mol/L BSA was injected into the microtubule for 30 min and rinsed by deionized water.

By using self-polymerization of dopamine in alkaline solution, a PDA layer with rich functional groups and strong adhesion was coated on the silicon surface of microtubule, which is suitable for antibody immobilization [46–48].

**Figure 3:**
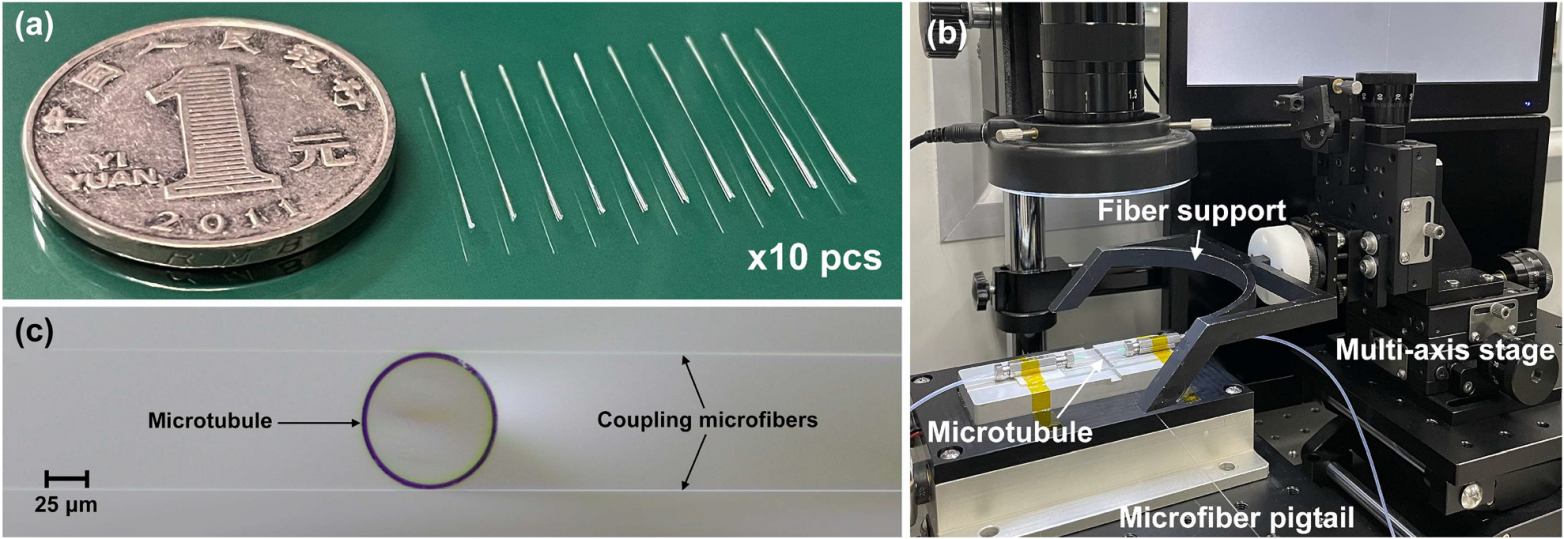
Coupling of disposable microtubule. (a) Photograph of the prepared disposable microtubules with inherent microfluidic channels for cTnI-C sample injection and detection. (b) Photo of the coupling platform for the double-fiber-coupled WGM microtubule cavity. (c) Optical microscopic image of the double-fiber-coupled WGM microtubule cavity.

Figure 2(c) and (d) show the scanning electron microscope (SEM) images of the untapered capillary and the tapered capillary with pressurization, owing to the air inflation for tapering microtubule, the wall of the capillary is reduced from 31.5 to 3 μm, while the wall of the tapered capillary without pressurization is 12 μm as shown in Figure 2(e). Figure 2(f) and (g) give the optical and fluorescence microscopic images of the microtubule immobilized FITC-conjugated cTnI antibody. Under the stimulated light, the green fluorescence emission could be clearly observed, validating the immobilization of cTnI antibody on the functionalized microtubule. Figure 3(a) gives the photograph of the prepared disposable microtubules with inherent microfluidic channels for cTnI-C sample injection and detection, the simple microtubules manufactured easily are disposable after biomarker detections, which helps to reduce the diagnosis cost and improve the diagnosis efficiency. Figure 3(b) shows the photo of the coupling platform for WGM microtubule cavity. The microfiber fixed on the metal support was adjusted close to the microtubule with a multi-axis stage, while the WGM resonance spectrum was observed through a spectrometer. In order to maintain the long-term stability of the fiber-coupled microtubule, the microfibers were bonded with the microtubule through a certain pressure, and fixed on a glass plate with low refractive index UV-curing adhesive. Figure 3(c) shows the optical microscopic image of the double-fiber-coupled WGM microtubule cavity. Furthermore, the ambient conditions including temperature and vibration isolation are strictly controlled to eliminate cross-sensitivity.

**Figure 4:**
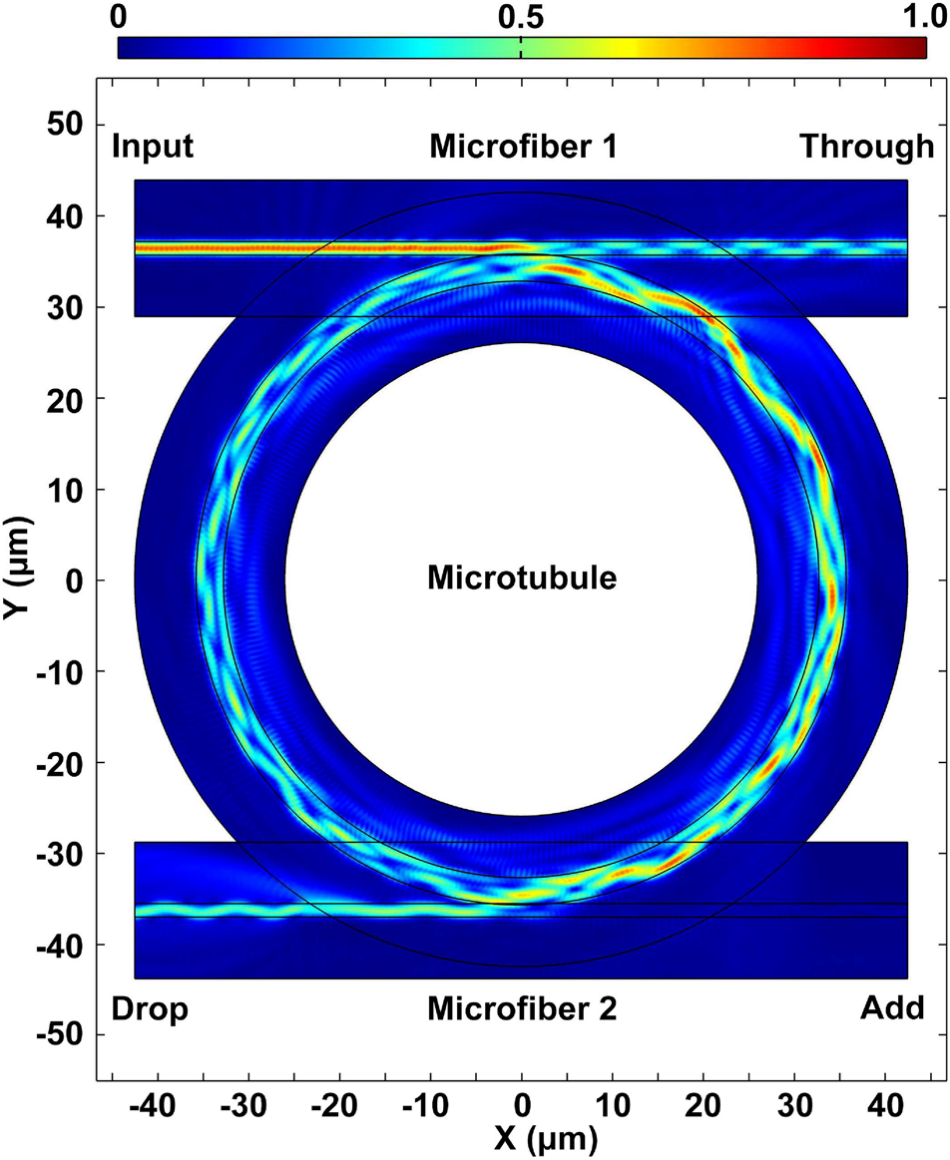
Simulated electric field distribution of the double-fiber-coupled microtubule cavity.

**Figure 5:**
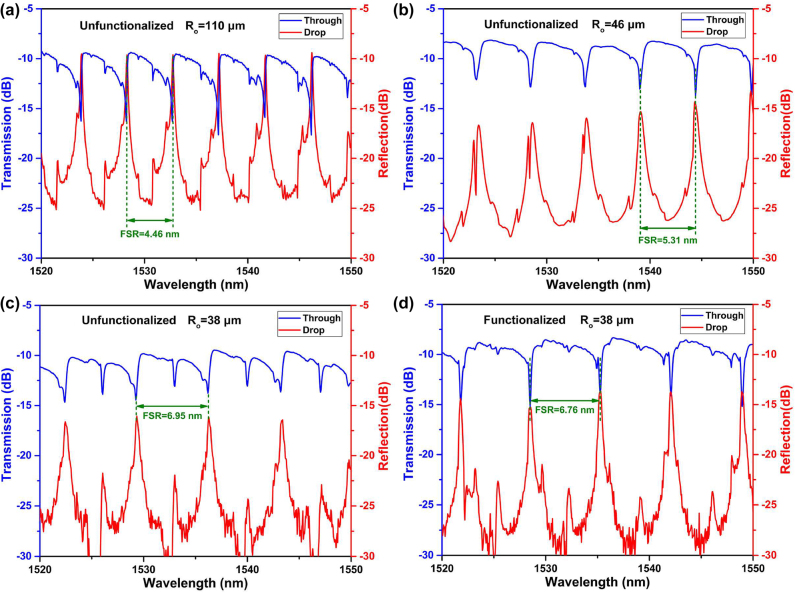
Excitation of WGM in double-fiber-coupled microtubule. Transmission and reflection spectra of the double-fiber-coupled microtubule cavities with (a) unfunctionalized *R*_o_ = 55 μm, (b) *R*_o_ = 46 μm, (c) *R*_o_ = 38 μm and (d) functionalized *R*_o_ = 38 μm.

In order to intuitively describe the filtering characteristics of microcavity, the electric field distribution of the double-fiber-coupled microtubule is simulated under different transmission and coupling efficiencies (Supplementary material, Section 1). Figure 4 shows the electric field distribution of the double-fiber-coupled microtubule cavity. In addition, the unfunctionalized double-fiber-coupled microtubule cavities with outside radii *R*_o_ = 55 μm, 46 and 38 μm were fabricated, and the transmission and reflection spectra are shown in Figure 5(a)–(d). It can be seen from the calculation and experimental results that the transmission and reflection spectra have the same resonant period, but the opposite spectral pattern. The resonant period, that is, the free spectral range FSR = *λ*_m_^2^/2π*Rn*_eff_ increases with decreasing microtubule radius, where *λ*_m_ is the resonant wavelength. The filtering output from the drop has a series of unique periodic resonant peaks and is suitable for laser wavelength selection. Furthermore, due to the PDA layer increases the *n*_eff_, the functionalization for microtubule narrows the FSR slightly, as shown in Figure 5(d), it could be illustrated that the immunological modification layer was effectively anchored onto the microtubule. When liquid containing cTnI-C is injected into the microtubule, the cTnI-C as antigen will be specifically captured by cTnI antibodies immobilized on the microtubule’s inner wall, and the more binding events of cTnI-C antigen–antibody will occur with the increasing cTnI-C concentration. Eventually, the binding of antigen–antibody leads to the increase of *n*_eff_ and the wavelength shifts of resonant peaks from drop port. Consequently, by using the filtering feature of the double-fiber-coupled microtubule cavity, the microcavity can be simultaneously employed as a sensing element with microfluidic channel and a wavelength selector to construct a FRL for the quantitative MI biomarker cTnI-C detection.

### 2.3 Active interrogation with lasing enhancement

**Figure 6:**
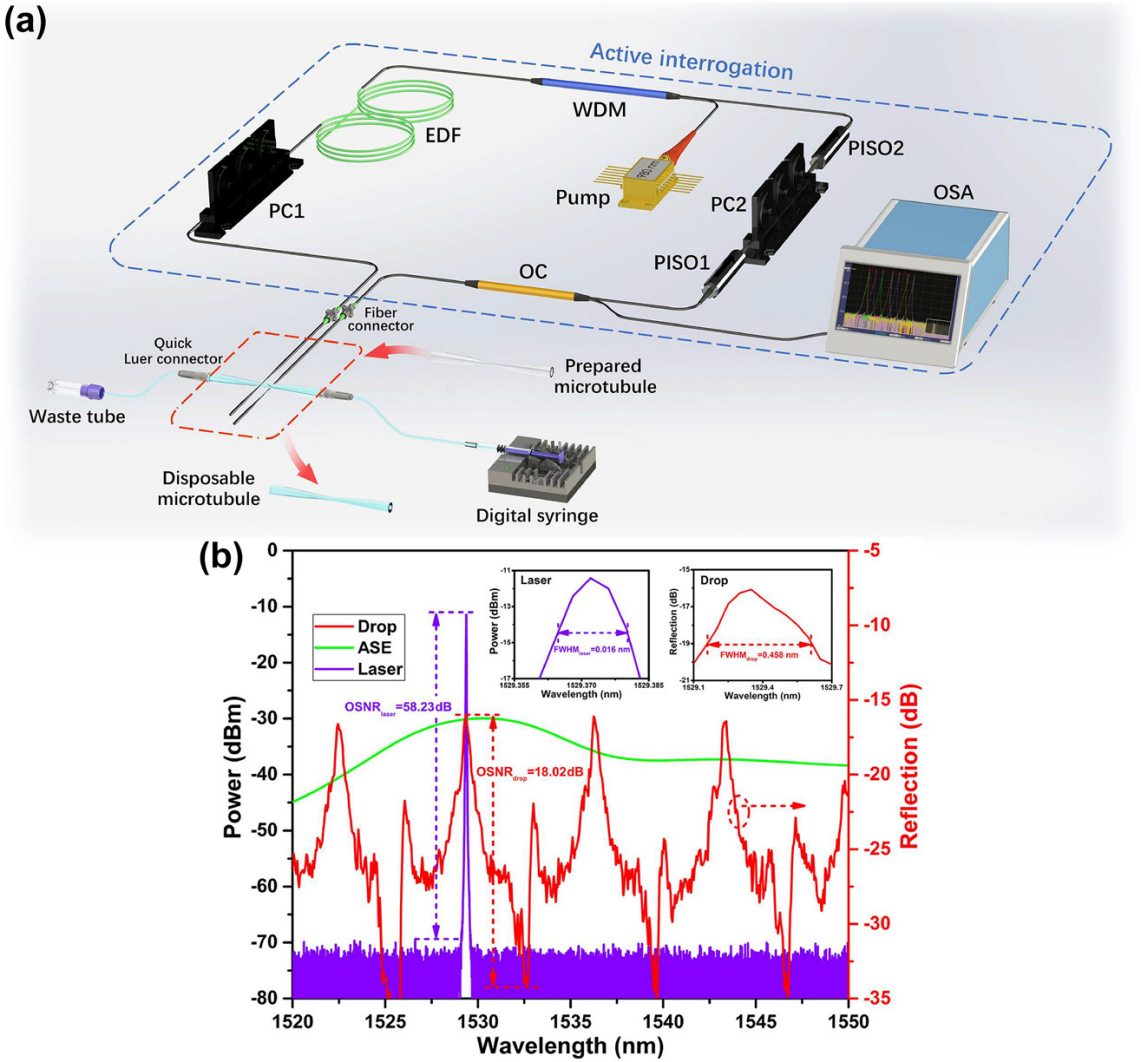
Active interrogation with lasing enhancement. (a) Schematic diagram of active interrogation for MI biomarker detection. WDM, wavelength division multiplexer; EDF, erbium-doped fiber; PC, polarization controller; OC, optical coupler; PISO, polarization-dependent isolator. (b) Forward-pump ASE and output sensing laser spectra of the FRL, and reflection spectrum from drop port of microtubule cavity. Insets: enlarged views of the peaks in the sensing laser and the original drop port.

Active interrogation can be implemented by embedding the MI biomarker sensitive microtubule cavity into FRL cavity, which has the merits of narrow spectral width and high optical power for high resolution detection. Although the active microcavity can provide sensing laser directly [49], the doping and coating of gain materials in the microcavity are complex and difficult to control, and the sensing laser power is restricted for high-sensitive sensing because of the limited gain in the microcavity. Through the high external-cavity gain in Er-doped FRL, active interrogation with high quality sensing laser can be obtained conveniently. The schematic diagram of active interrogation for MI biomarker detection is shown in Figure 6(a). The gain of the FRL is provided by a section of 1 m erbium-doped fiber (EDF, Er80-4/125, Liekki), which is pumped by a 980 nm laser source with 980/1550 nm wavelength division multiplexer (WDM). Two polarization-dependent isolators (PISO1 and PISO2) and two polarization controllers (PC1 and PC2), are used to support two mutual-coupled polarimetric loops in a single physical FRL cavity, and then a stable single-wavelength sensing laser can be generated with parity-time symmetry breaking [50, 51]. The output sensing laser for MI biomarker detection is extracted by a 5:95 optical coupler (OC), and recorded by an optical spectrum analyzer (OSA, AQ6370, Yokogawa). A semiconductor thermostat was used to maintain a constant experimental temperature of 26 °C, and the cTnI-C samples under test were injected into the microtubule by a digital syringe pump with a fixed flow rate of 10 μL/min. Smaller radius of microtubule could broaden the FSR and facilitate lasing wavelength selection, but at the same time it will reduce the flow of samples and reduce the mechanical strength of the sensing element. Considering the mechanical strength and sample flux of the microcavity, a functionalized microtubule cavity with *R*_o_ = 38 μm was used in experiment.

Figure 6(b) gives the forward-pump amplified spontaneous emission (ASE) and output sensing laser spectra of the FRL. By adjusting the polarization states of light in the single spatial FRL cavity with two polarization controllers, the wavelength-dependent gain unevenness was regulated within the gain bandwidth, and the corresponding resonant peak of the WGM obtained the cyclic-cumulative gain and be amplified in the FRL cavity. Eventually, the stimulated emission was generated at the resonant peak wavelength with maximum gain. Compared with the original reflection from the drop port, the single-wavelength output from the FRL possesses narrower FWHM of 0.016 nm and higher OSNR of 58.23 dB, as shown in the insets of Figure 6(b), which helps to improve the detection limit for biomarker sensing. The measured FWHM is limited by the wavelength resolution of the OSA, and the smaller FWHM can be obtained by the beat frequency method [52]. In our experimental configuration, the OSA is directly used for efficient interrogation. Thus, the cTnI-C detection can be easily achieved by interrogating the lasing wavelength of the high-quality sensing laser from FRL. Because the spatial filtering feature of WGM cavity is related to the cTnI-C concentration in the disposable sample microtubule, the lasing wavelength of output sensing laser from FRL can be tuned by the cTnI-C concentration of injected liquid sample.

## 3 Results and discussion

### 3.1 Responses to refractive index and temperature

**Figure 7:**
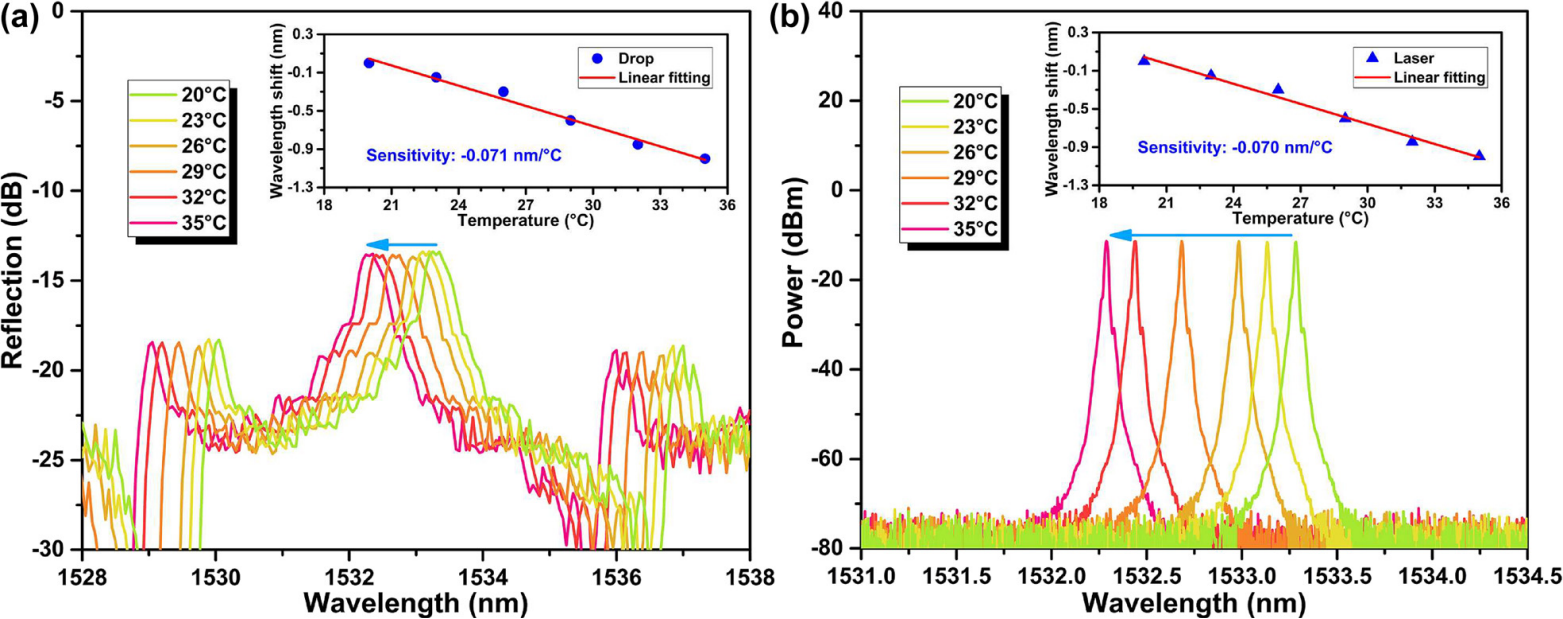
Spectral evolutions of (a) the drop port reflection and (b) the output sensing laser at different temperatures. Insets: temperature sensitivities of peak wavelengths.

Cross-sensitivity is one of the important factors affecting biosensor’s performance, characterizing the response of biosensor to environmental variations is helpful to improve the practicability of measurement. The maintaining temperature of the WGM microtubule cavity was changed from 23 °C to 35 °C with an interval of 3 °C. The spectral evolutions of the drop port reflection and the output sensing laser at different temperatures were recorded by OSA, as depicted in Figure 7(a) and (b), respectively, and the insets of Figure 7(a) and (b) shows the sensitivities of peak wavelengths to temperature. With the increase of temperature, the drop port reflection shifts to the shorter wavelength, and the linear fitting shows a temperature sensitivity of −0.071 nm/°C. Because the output sensing laser wavelength is determined by the drop characteristics of the WGM microtubule cavity, the peak wavelength of sensing laser has the same temperature shift direction and a similar temperature sensitivity of −0.070 nm/°C.

Considering that the temperature accuracy of the semiconductor thermostat used in experiments is ±0.01 °C, the wavelength shift caused by the possible temperature fluctuation is ±7.1 × 10^−4^ nm, and therefore the resulting measurement error for cTnI-C is 1.05 × 10^−4^ ng/mL, which is less than the estimated detection resolution of 1.48 × 10^−4^ ng/mL. Since biomarker detection should be a statistical result of dynamic process, the impact of ambient fluctuation can be compensated by multiple measurements with comprehensive statistical analysis.

**Figure 8:**
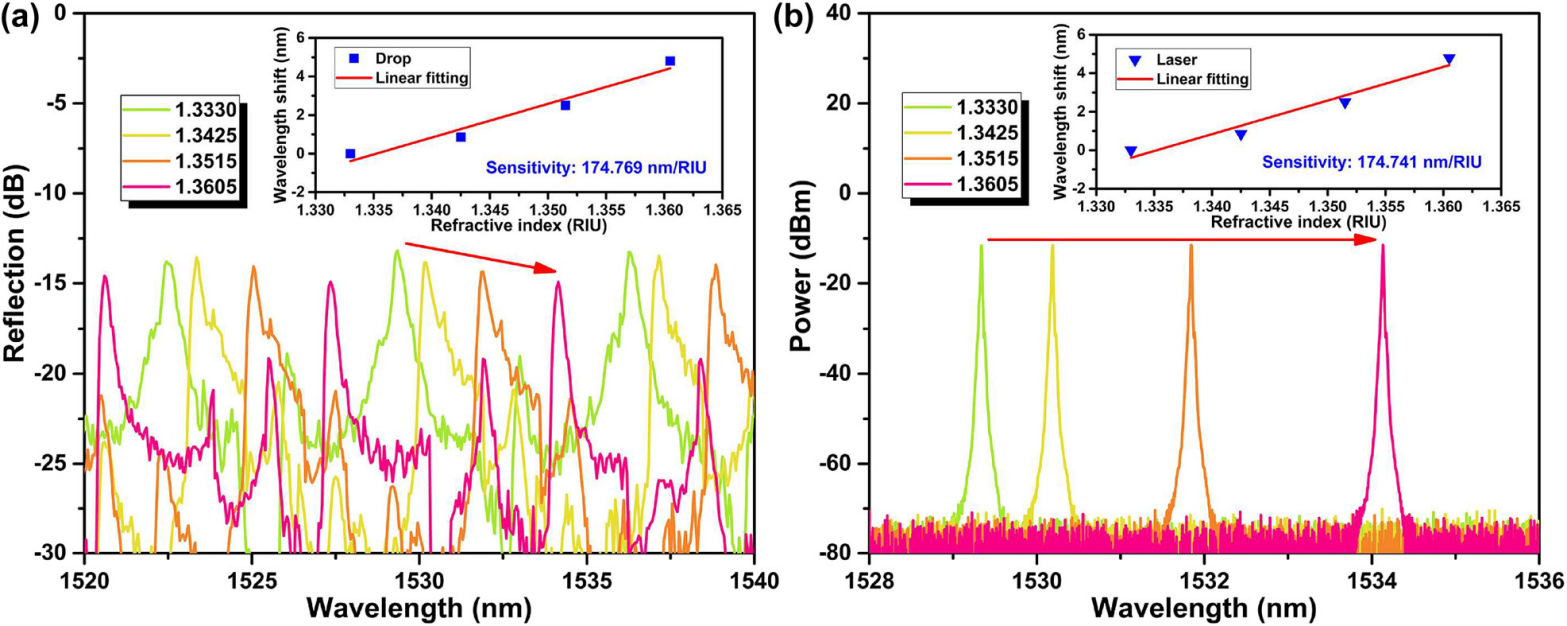
Spectral evolutions of (a) the drop port reflection and (b) the output sensing laser at different refractive index. Insets: refractive index sensitivities of peak wavelengths.

To investigate the refractive index sensitivity of the WGM microtubule cavity, a series of liquid samples were prepared by diluting the NaCl solution with deionized water, which were calibrated by Abbe refractometer. After the liquid samples were successively injected into microtubule, the spectra of the drop port and the sensing laser at different refractive indexes were measured as shown in Figure 8(a) and (b), and the sensitivities of peak wavelengths to refractive index are plotted by linear fitting in the insets of Figure 8(a) and (b). As the surrounding refractive index in microtubule increasing, the drop reflection and sensing laser have blueshifts. The sensitivities to refractive index are 174.769 nm/RIU and 174.741 nm/RIU during 1.3330–1.3605. In addition, the wavelength shifts of the through and drop spectra and the sensing laser were simulated at different refractive indexes (Supplementary material, Section 2). The simulation and experimental results verify the refractive index characteristics of the WGM microtubule cavity and the feasibility of the active interrogation for biomarker detection.

Table 1 summarizes sensitivities of the proposed WGM microtubule cavity and the optical fiber sensors based on evanescent field. Although microfiber-based optical sensors generally have ultra-high refractive index sensitivities, the fragile microfiber is not suitable for biochemical sensing in high-throughput fluid environment. Compared with optical fiber SPR structures and mode interferometers, the proposed microtubule cavity has comparable sensitivity and inherent microfluidic channel, which is easy to be fabricated without metal film deposition and fusion splicing.

**Table 1:** A comparison between the proposed WGM microtubule cavity and the optical fiber sensors based on evanescent field.

Scheme	Sensitivity (nm/RIU)	Sensitivity (nm/°C)	Cross-sensitivity (RIU/°C)	Ref.
Microfiber grating	1534.78	1.033 × 10^−2^	6.7 × 10^−6^	[53]
Microfiber coupler	586.74	0.44366	7.6 × 10^−4^	[54]
Microfiber knot resonator	116	5.6 × 10^−3^	4.8 × 10^−5^	[55]
Metal film SPR	1174	0.7	6.0 × 10^−4^	[56]
Multimode interferometer	135.47	1.88 × 10^−2^	1.4 × 10^−4^	[57]
Mach–Zehnder interferometer	135.31	−3.897 × 10^−2^	2.9 × 10^−4^	[58]
Fabry–Perot interferometer	5.49	1.6 × 10^−3^	2.9 × 10^−4^	[59]
WGM microtubule cavity	174.769	−7.1 × 10^−2^	4.1 × 10^−4^	This work

### 3.2 Static response to cTnI-C complex

**Figure 9:**
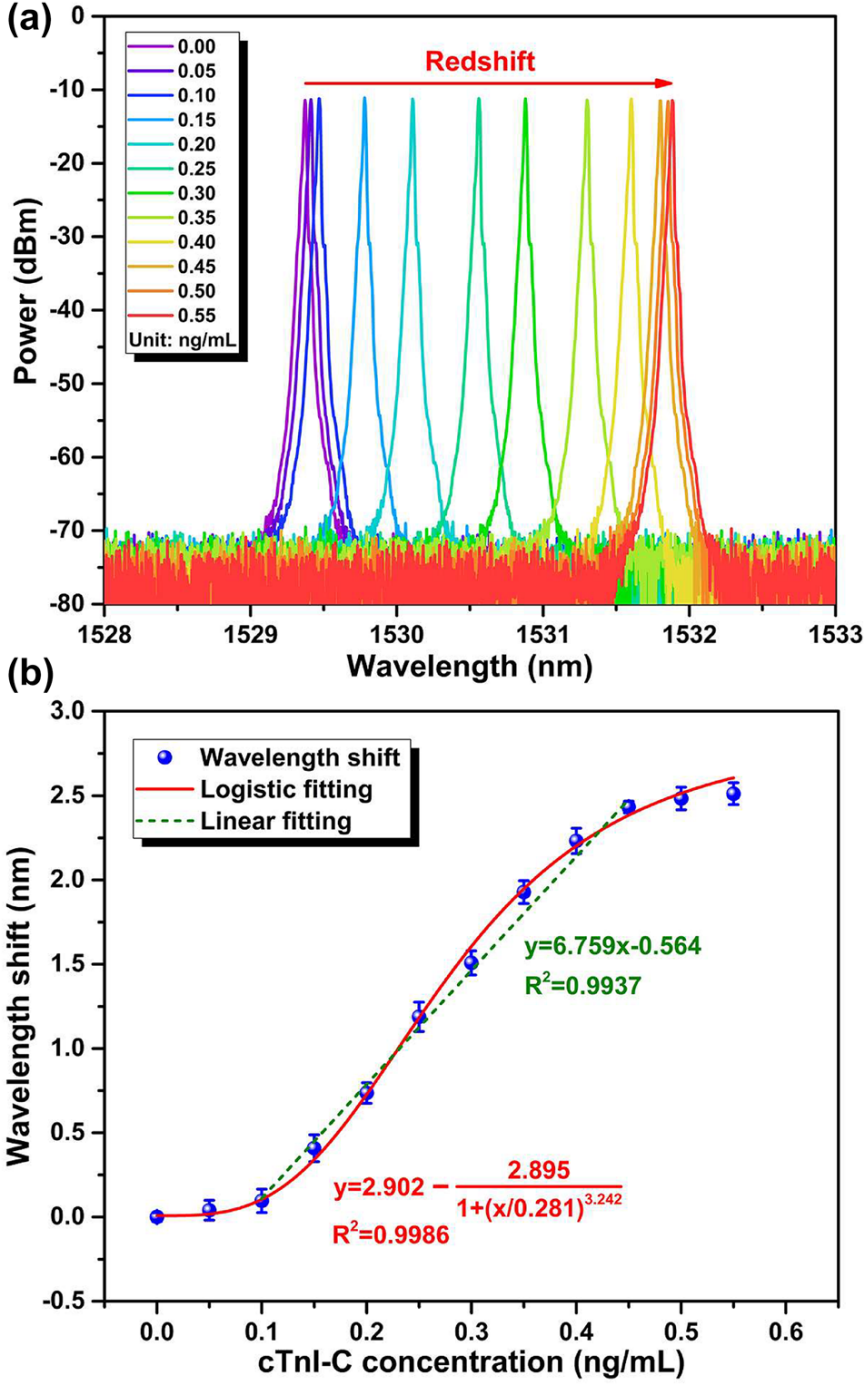
Experimental results of static response. (a) Spectrum evolution of the output sensing laser from the FRL with increasing cTnI-C concentration. (b) Response of the peak wavelength shift of the sensing laser to the cTnI-C concentration.

Figure 9(a) shows the spectrum evolution of the output lasing spectra with increasing cTnI-C concentration. With the increase of the cTnI-C concentration, the sensing laser wavelength shifts to the longer wavelength, because the binding events of cTnI-C antigen–antibody occur on the inner wall surface of the microtubule cavity, and the effective refractive index of the microtubule waveguide is increased. For the initialization of detection, the microtubule cavity was flushed by using the mild protein elution buffer of glycine-HCl [60, 61], and the elution buffer was injected into the microtubule with real-time monitoring of the sensing laser wavelength to be the initial. At each concentration, the peak wavelength of the sensing laser was repeatedly measured 10 times after 30 min detection. As shown in Figure 9(b), the output sensing laser shifts 2.512 nm during the range of 0.00–0.55 ng/mL, and the response curve of the peak wavelength shift to cTnI-C concentration satisfies a logistic fitting as follow [62]:

(5) $$y=2.902-\frac{2.895}{1+{\left(x/0.281\right)}^{3.242}}$$

the LOD of the proposed microtubule cavity can be calculated as low as 0.137 ng/mL [63]. The linear fitting results in Figure 9(b) show the wavelength sensitivity is 6.759 nm/(ng/mL) in partial range of 0.10–0.45 ng/mL.

In order to evaluate the sensing performance of the microtubule cavity, the DL and the quality factor *Q* were introduced [34, 35], which indicate the sensing properties of optical fiber sensors with consideration of spectral quality. A feature comparison between the original and the FRL enhanced microtubule cavities is listed in Table 2. Owing to the spectral bandwidth compression and power amplification of the FRL configuration, the characteristic resonant peak for cTnI-C detection was enhanced in FWHM and OSNR, and thus the DL and *Q* were accordingly improved without sacrificing sensitivity. Moreover, Table 3 gives a performance comparison between the proposed WGM optofluidic microcavity and the other optical fiber biosensors for cTnI detection. Compared with the SPR and microfiber FBG based sensors, the proposed microtubule cavity provides higher sensitivity and smaller resolution, the DL and *Q* are enhanced by up to 4 and 5 orders of magnitude. Although the biosensor based on microfiber coupler has a higher sensitivity, the limited concentration range within 10 fg/mL is too narrow to apply for diagnosis, and the sensing spectra with smooth resonant peaks also limit its performances. For the proposed microtubule cavity, the demonstrated detection range of 0.137–0.55 ng/mL can meet the requirement of early-stage MI diagnosis [64]. Compared to plasmonic nanostructure and metasurface biosensors (Supplementary material, Section 3) [65, 66], the proposed all-fiber scheme simplifies the manufacturing process and reduces costs while maintaining relatively high sensitivity and *Q*-factor, allowing for better compatibility with fiber-optic systems and eliminating redundant optical components. Therefore, the proposed microtubule cavity with FRL enhancement provided a fiber-optic integrated method for cTnI-C detection, which could be a diagnostic tool for MI.

**Table 2:** A feature comparison between the original and the FRL enhanced microtubule cavities.

Parameters	Original	FRL enhanced	Enhancement
FWHM	0.458 nm	0.016 nm	28.63
OSNR	18.02 dB	58.23 dB	40.21 dB
DL^*^	0.016	1.40 × 10^−4^	114.29
Q^*^	1.80 × 10^3^	1.66 × 10^5^	92.22

^*^At the same optical wavelength resolution of 1 pm.

**Table 3:** A performance comparison between the proposed WGM optofluidic microcavity and the other optical fiber biosensors for cTnI detection.

Scheme	FWHM	OSNR	DL^**^	*Q* ^**^	Sensitivity	Resolution^**^	LOD	Concentration range	channel	Refs.
	(nm)	(dB)	(ng/mL)		(nm/(ng/mL))	(ng/mL)	LOD (ng/mL)	(ng/mL)	channel	
SPR multi-mode fiber	>60^*^	<3^*^	>1^*^	<1^*^	3 × 10^−3*^	0.33^*^	1.4	0–100	None	[26]
Optical microfiber coupler	>5^*^	<3^*^	>1^*^	<5 × 10^5*^	900^*^	1.11 × 10^−6*^	2 × 10^−3^	0–0.01	None	[27]
Phase-shifted mFBG	0.15	<15^*^	0.42^*^	<1^*^	0.1^*^	0.01^*^	0.03	0–100	None	[28]
Harmonic mFBG	>2	<20^*^	>1^*^	<1^*^	0.01^*^	0.1^*^	13.5	0–1000	None	[29]
This work	0.016	58	1.40 × 10^−4^	1.66 × 10^5^	6.759	1.48 × 10^−4^	0.137	0–0.55	Possessed	

^*^The calculation results were estimated according to the optimal data in the references.

^**^At the same optical wavelength resolution of 1 pm.

### 3.3 Real-time monitor of the dynamic binding and unbinding process of cTnI-C antigen–antibody

**Figure 10:**
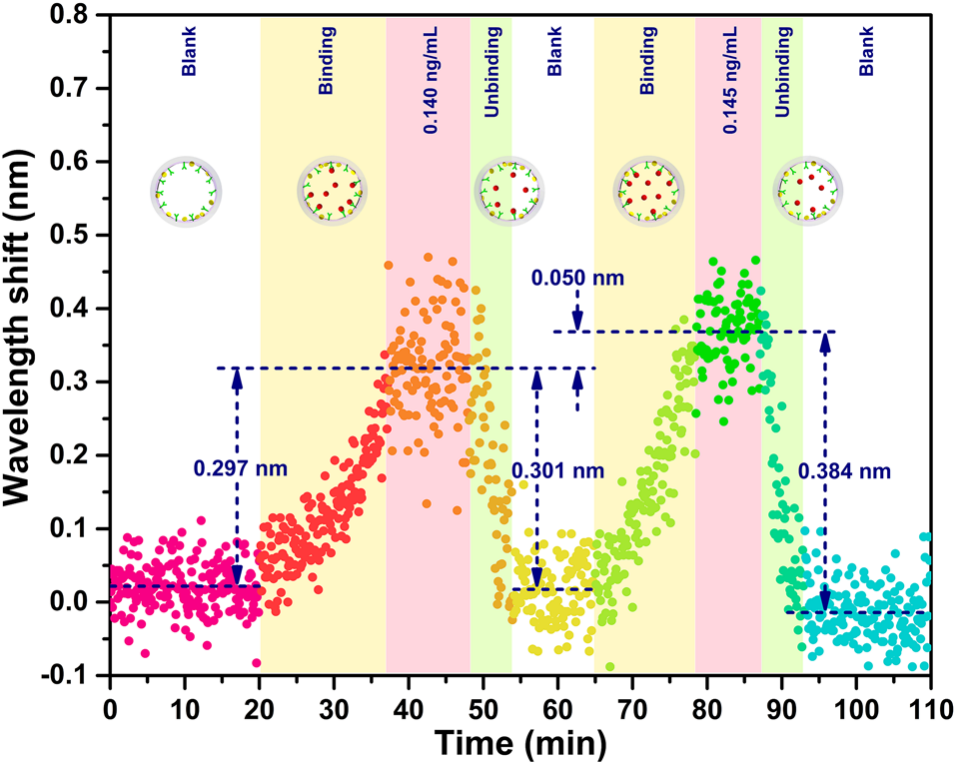
Dynamic response of the peak wavelength shift of the sensing laser to the binding and unbinding process of cTnI-C antigen–antibody.

The binding and unbinding of cTnI-C antigen–antibody is a dynamic process, and the real-time monitor of this process is relevant to evaluate the avidity and reaction state of the antigen and antibody. The proposed optofluidic microtubule immunosensor has the ability to monitor the biomolecular binding events in real-time through effective refractive index changes near the inner wall surface of microtubule. To describe the binding and unbinding process of cTnI-C antigen–antibody, the dynamic response of the sensing laser to cTnI-C with the concentrations of 0.140 ng/mL and 0.145 ng/mL near the LOD, was investigated by measuring the lasing peak wavelength shift continuously, as shown in Figure 10. After 20 min of standby state with blank PBS, the 0.140 ng/mL cTnI-C sample was injected into the microtubule, and the lasing peak wavelength immediately and gradually shifts owing to the binding process of cTnI-C antigen–antibody, the binding process lasted ∼17 min and the lasing peak wavelength reaches a plateau with an average shift of 0.297 nm. Subsequently, the elution buffer was injected to dissociate the cTnI-C antigens and antibodies. With the unbinding process of cTnI-C antigen–antibody, the lasing peak wavelength quickly returns to the blank state. After 10 min of PBS flushing, the cTnI-C sample with a slightly higher concentration of 0.145 ng/mL was injected, and an average lasing peak wavelength shift of 0.301 nm occurs indicating the binding of cTnI-C antigen–antibody is saturated. The wavelength shift difference of 0.050 nm between the two samples with a small concentration difference of 0.005 ng/mL is consistent with the response curve in Figure 9(b). The experimental results of the dynamic response demonstrate that the high-throughput microfluidic channel of the microtubule provides a fast response time of ∼15 min for emergency diagnosis of MI, and the proposed optofluidic microtubule immunosensor possesses fast response, high-sensitivity, stability and regeneration in cTnI-C detection, it can be an efficient tool for the real-time monitor of biomolecular reaction process and the biomolecular affinity assessment.

### 3.4 Specificity

**Figure 11:**
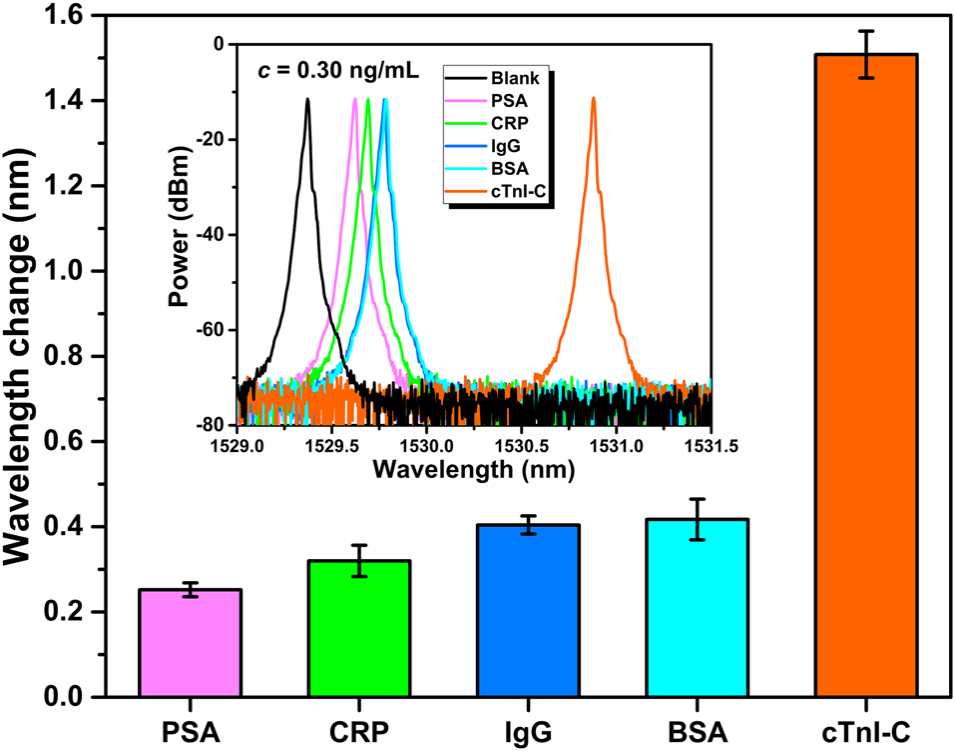
Peak wavelength changes of sensing laser with the interference and cTnI-C samples for specificity test (PSA: prostate specific antigen; CRP: C-reactive protein; IgG: immunoglobulin G; BSA: bovine serum albumin). Inset: the corresponding sensing laser spectra. Each sample was injected into to the microtubule cavity at the same concentration of 0.3 ng/mL in PBS buffer.

The detecting specificity of the WGM microtubule cavity was tested by employing the four kinds of non-specific interference samples prostate specific antigen (PSA), C-reactive protein (CRP), immunoglobulin G (IgG) and BSA, and the employed interference and cTnI-C samples were diluted in PBS with a same concentration of 0.3 ng/mL. The peak wavelength changes of sensing laser with the interference and cTnI-C samples are shown in Figure 11, and the inset of Figure 11 gives the corresponding sensing laser spectra. The redshift of laser wavelength for the target cTnI-C was evidently more than those of the four interference samples at the same concentration, which indicated that the developed microtubule cavity with stable PDA functionalization had high selectivity for the cTnI-C detection.

## 4 Conclusions

We have proposed and demonstrated a label-free cTnI-C immunosensor based on disposable optofluidic microtubule with active interrogation enhancement. The thin-wall microtubule cavity with inherent microfluidic channel is simply fabricated by the silica capillary with pressurized tapering technology. By using the self-adherent PDA, the cTnI antibodies are anchored onto the inner wall of the microtubule for detecting specificity. With the double-fiber coupling configuration, the microtubule cavity as a tunable filter is introduced into a polarimetric FRL, and the spectral width of the resonant peak in optical sensing signal is compressed to 0.016 nm and the OSNR is as high as 58.23 dB. During the concentration range of 0.05–0.55 ng/mL, the optical immunosensor for cTnI-C is achieved by measuring the output laser wavelength of the FRL conveniently, and a LOD of 0.137 ng/mL is obtained by a logistic fitting. By continuously measuring the lasing peak wavelength, the real-time monitor of the dynamic binding and unbinding process of cTnI-C antigen–antibody is demonstrated experimentally with a fast response time of ∼15 min for emergency diagnosis of MI. With the merits of fast label-free detection, real-time monitor and flexible usage, the analytical performance of our immunosensor indicated its great prospect for bioanalysis and clinical diagnosis of AMI, and the disposable all-fiber immunosensor would enlighten the development for high-sensitive and high-efficient optofluidic biosensors based on optical fiber laser and sensing technologies to MI biomarker detection.

## Supplementary Material

Supplementary Material Details
