# Supplementary material for: High-sensitive and disposable myocardial infarction biomarker immunosensor with optofluidic microtubule lasing
Source: Nanophotonics. 2022 Jun 7;11(14):3351–64. doi: 10.1515/nanoph-2022-0208 (PMC11501927; doi:10.1515/nanoph-2022-0208)
Supplement: Supplementary file 1 — Supplementary Material Details [file j_nanoph-2022-0208_suppl.docx]

High-sensitive and disposable myocardial infarction biomarker immunosensor with optofluidic microtubule lasing

Supplementary Material

Panpan Niu^1,2,3^, Junfeng Jiang^1,2,3,*^, Kun Liu^1,2,3^, Shuang Wang^1,2,3^, Tong Wang^1,2,3^, Yize Liu^1,2,3^, Xuezhi Zhang^1,2,3^, Ding Zhenyang^1,2,3^, Tiegen Liu^1,2,3^

^1^School of Precision Instrument and Opto-electronics Engineering, Tianjin University, Tianjin 300072, China

^2^Key Laboratory of Opto-electronics Information Technology (Tianjin University), Key Laboratory of Micro Opto-electro Mechanical System Technology (Tianjin University), Ministry of Education, Tianjin 300072, China

^3^Tianjin Optical Fiber Sensing Engineering Center, Institute of Optical Fiber Sensing of Tianjin University, Tianjin 300072, China

* jiangjfjxu@tju.edu.cn

**Contents:**

**Section 1. Electromagnetic simulation of double-fiber-coupled microtubule cavity**

**Section 2. Numerical estimation of sensing laser**

**Section 3. A summary comparison between the proposed optical fiber biosensor and the recent plasmonic nanostructures and metasurfaces-based optical biosensors.**

**Section 1. Electromagnetic simulation of double-fiber-coupled microtubule cavity**

In our work, the whispering gallery modes (WGMs) in microtubule are excited by using coupled microfiber, and the coupling efficiency between microtubule and microfiber is important for the generation of WGM and sensing laser. Because the light coupling is realized by surface evanescent field, the coupling efficiency can be controlled by changing the diameter of microfiber (thinner microfiber provides stronger evanescent field) and the distance between microfiber and microtubule. In order to optimize the coupling efficiency, the coupling is simulated by simplifying the microfiber and the microtubule into straight waveguide and ring waveguide. In the finite element model, the diameters of the microfiber and the microtubule are 1.5 μm and 73 μm, respectively, the wall thickness of the microtubule is 3 μm. The surrounding medium of microfiber and microtubule is air with refractive index *n*_air_=1, the medium in microtubule is water with *n*_air_=1.3330, the refractive indexes of microfiber and microtubule are *n*_fiber_=1.4682 and *n*_tube_=1.49, respectively. The wavelength of input light field is 1550 nm. The coupling distance *D*_cp_ between microfiber and microtubule is gradually reduced from 0.45 μm to −1.65 μm, and the negative distance value indicates the fit degree after the optical fiber is in contact with the microtubule under a certain pressure.

Fig. S1 gives the calculated transmission efficiency and coupling efficiency of coupling point 1 (microfiber into microtubule) with *D*_cp_ range of 0.45 μm~1.65 μm. Fig. S2 shows the electric field distributions of the coupling point 1 with *D*_cp_= 0.45 μm, 0.15 μm, 0 μm, −0.15 μm, −0.30 μm, −0.90 μm, −1.35 μm and −1.65 μm. Fig. S3 gives the electric field distributions of the double-fiber-coupled microtubule cavity with *D*_cp_= 0.45 μm, 0.15 μm, 0 μm, −0.15 μm, −0.30 μm, −0.90 μm, −1.35 μm and −1.65 μm. The simulation results show that, the transmission efficiency decreases with *D*_cp_ decreasing, and the coupling efficiency increases, more light energy is coupled from the microfiber into the microtubule, the electric field intensity of the through port decreases. When *D*_cp_ decreases to −0.4 μm~−0.3 μm, the coupling efficiency is the highest. However, if the *D*_cp_ continues to decrease, the coupling efficiency decreases and the electric field intensity of through port increases. Therefore, the *D*_cp_ and fit degree between the optical fiber and the microtubule should be optimized, it can be implemented by monitoring the drop port spectrum during the adjusting process of *D*_cp_ in the experiment. The appropriate pressure applied to the microfiber will increase coupling area, which is conducive to improve the coupling efficiency and the stability of the coupling structure. In addition, the low coupling efficiency in the simulation results is caused by the simplified simulation model based on planar waveguide.


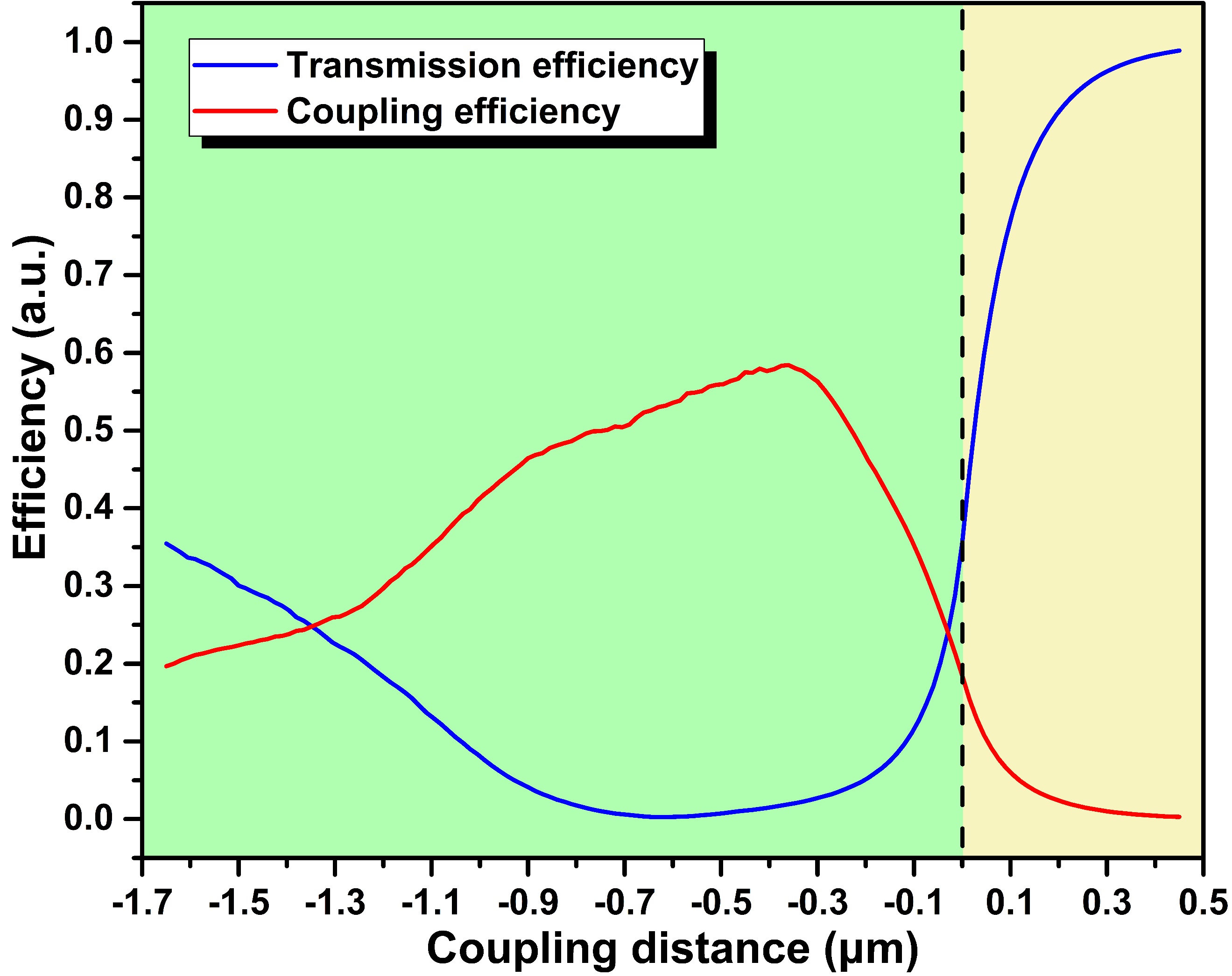


Fig. S1. Calculated transmission efficiency and coupling efficiency of coupling point 1 with *D*_cp_ range of 0.45 μm~1.65 μm.


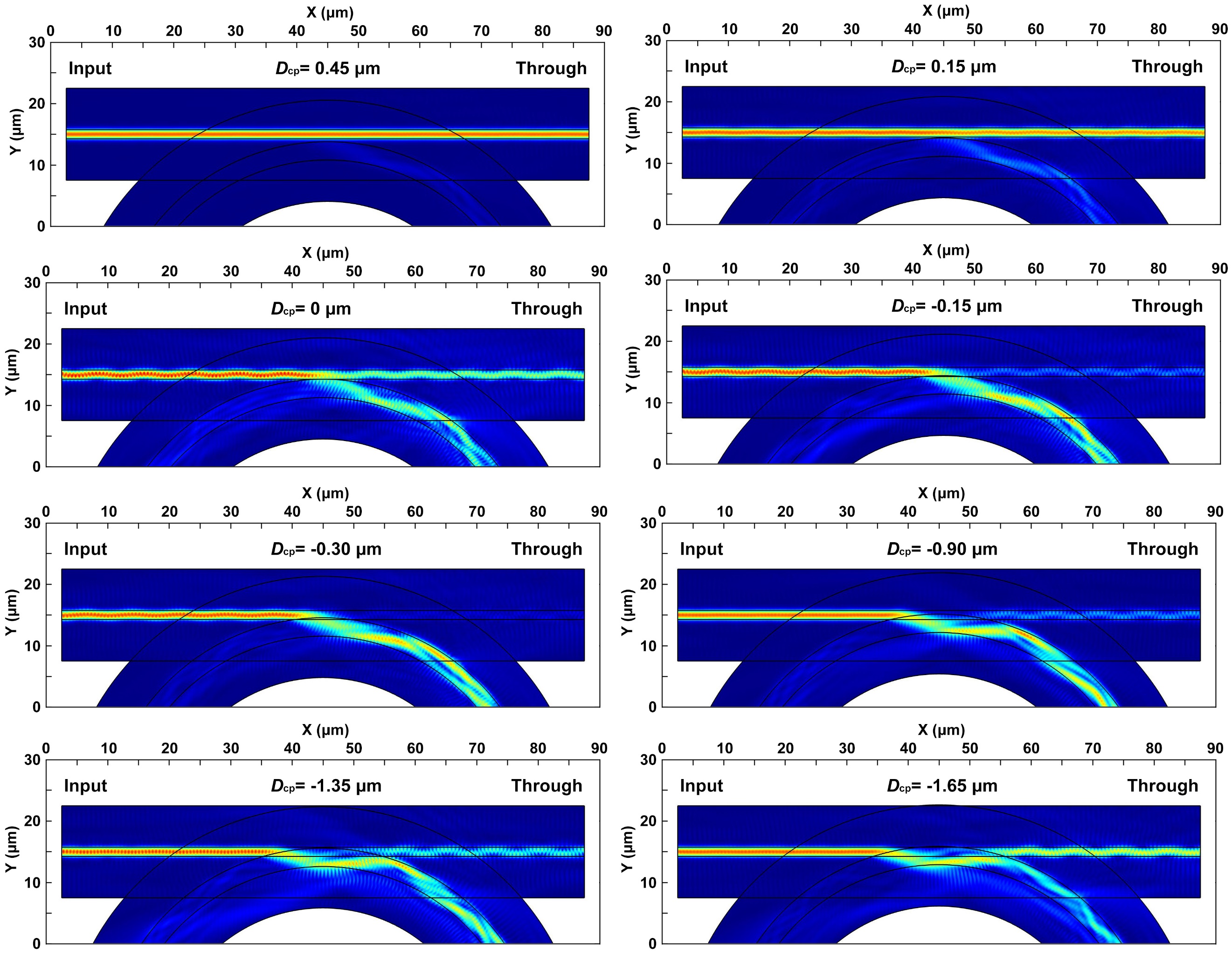


Fig. S2. Electric field distributions of the coupling point 1 with *D*_cp_= 0.45 μm, 0.15 μm, 0 μm, −0.15 μm, −0.30 μm, −0.90 μm, −1.35 μm and −1.65 μm.


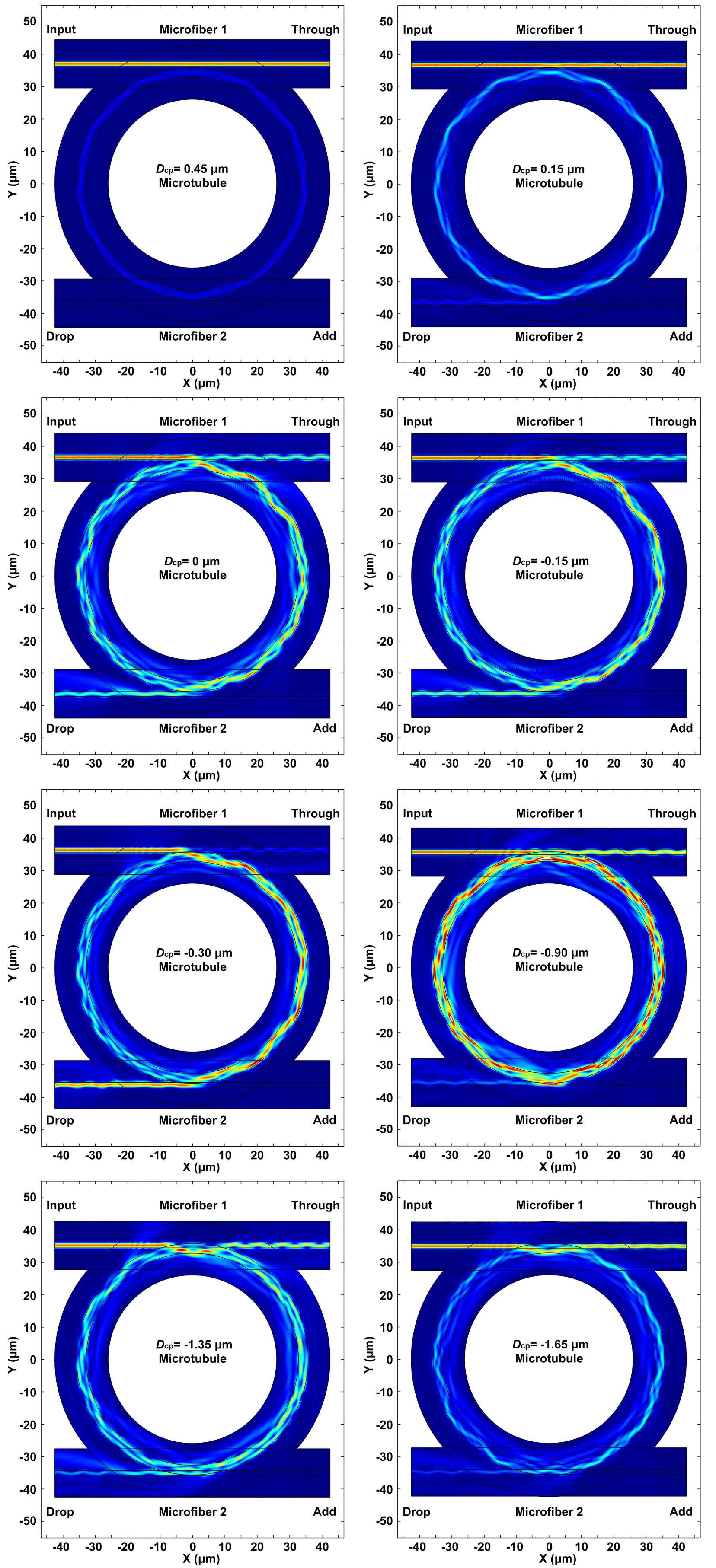


Fig. S3. Electric field distributions of the double-fiber-coupled microtubule cavity with *D*_cp_= 0.45 μm, 0.15 μm, 0 μm, −0.15 μm, −0.30 μm, −0.90 μm, −1.35 μm and −1.65 μm.

**Section 2. Numerical estimation of sensing laser**

*2.1 Energy level rate equation of Er^3+^*

Under the excitation of 980 nm pump light, Er^3+^ in rare earth -doped fiber has the condition of realizing particle number inversion, Er^3+^ behaves as a three-energy level system, as shown in Fig. S4. *E*_1_ (*n*_1_) is the ground state energy level, *E*_2_ (*n*_2_) is the metastable energy level (also known as the excited state energy level), *E*_3_ (*n*_3_) is the pump high energy level, *n*_1_, *n*_2_ and *n*_3_ are the numbers of particles in the ground state energy level, excited state energy level and pump high energy level, respectively. In the interaction between light and gain medium, *W* represents the probability of stimulated transition, *A* represents the probability of spontaneous emission transition, and *S* represents the probability of non-radiative transition.


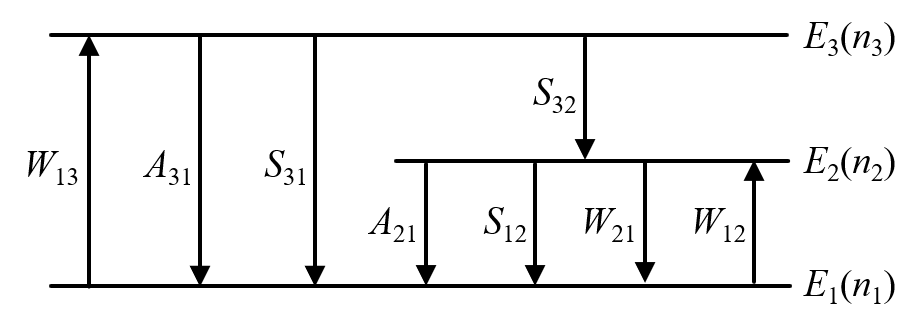


Fig. S4. Schematic diagram of the three-level energy system of Er^3+^.

Under the pump of light, the particles at *E*_1_ level absorb the pump light energy and transfer to *E*_3_ level through stimulated absorption. Due to the unstable state of the particles at *E*_3_ level, and the particles will quickly transfer to *E*_2_ level. This process shows a non-radiative transition. The particles at *E*_2_ level have a long life, they return to the ground state level mainly by spontaneous emission photons. The total probability of the spontaneous emission transition and non-radiative transition of the particles at *E*_2_ level is about the reciprocal of the lifetime of the particles at *E*_2_ level, *A*_21_+*S*_21_≈1/*τ*, and then the energy level rate equation of Er^3+^ can be obtained as follows:

$\frac{dn_{2}}{dt}=W_{12}n_{1}-W_{21}n_{2}-\frac{n_{2}}{\tau}$ (S1)

$n_{1}+n_{2}=n$ (S2)

where *W*_12_ is the probability of stimulated transition of particles at *E*_1_ level to *E*_2_ level, *W*_21_ is the probability of stimulated transition of particles at *E*_2_ level to *E*_1_ level, and *n* represents the total number of particles.

*2.2 Traveling wave rate equation of ring laser cavity*

For erbium-doped fiber ring laser cavity, the laser operates in the form of traveling wave, which can be described by a traveling wave rate equation as follow:

$\frac{dn_{2}}{dt}=\frac{\Gamma_{\text{p}}\sigma_{\text{pa}}P_{\text{p}}}{h\upsilon_{\text{p}}}n_{1}-\int\left(\sigma_{\text{se}}(\upsilon)n_{2}-\sigma_{\text{sa}}(\upsilon)n_{1}\right)\Gamma_{\text{s}}\frac{P_{ASE}^{+}(\upsilon)+P_{ASE}^{-}(\upsilon)}{h\upsilon}d\upsilon-\frac{n_{2}}{\tau}$ (S3)

$\frac{dP_{\text{p}}}{dz}=-\Gamma_{\text{p}}\sigma_{\text{pa}}n_{1}P_{\text{p}}$ (S4)

$\frac{dP_{\text{ASE}}^{\pm}(\upsilon)}{dz}=g(\upsilon)\left[P_{\text{ASE}}^{\pm}(\upsilon)+2n_{\text{sp}}h\upsilon\right]$ (S5)

$g(\upsilon)=\left(\sigma_{\text{se}}(\upsilon)n_{2}-\sigma_{\text{sa}}(\upsilon)n_{1}\right)\Gamma_{\text{s}}$ (S6)

$n_{\text{sp}}=\frac{n_{2}}{(1+\sigma_{\text{sa}}/\sigma_{\text{se}})n_{2}-(\sigma_{\text{sa}}/\sigma_{\text{se}})n}$ (S7)

where Г_P_ and Г_s_ is the overlapping integral factors of pump light and signal light with Er^3+^, σ_pa_, σ_se_ and σ_sa_ represent the absorption cross section for pump light, the emission cross section for signal light and the absorption cross section for signal light, respectively. For pump light with different frequencies, the absorption and emission cross sections are different. *P*_P_ and *P*_ASE_ is pump power and amplified spontaneous emission (ASE) light power, and the ± of *P*_ASE_ represent direction (forward and backward) of ASE. When there is no external signal light, the ASE in the ring cavity is the signal light. *υ*_P_ is the pump frequency, g(*υ*) is the gain coefficient, *n*_sp_ the inversion factor of particle number, and *h* is the Planck constant.

*2.3 Numerical solution of traveling wave rate equation*

The traveling wave rate equation of ring cavity is a set of coupled differential equations, which can be solved by the time-domain step-by-step method, as shown in Fig. S5. The erbium-doped fiber with length *L* is evenly divided into *K* segments, and the length of each segment is Δ*z*=*L*/*K*, the corresponding light propagation time is Δ*t*=Δ*z*/*v*_g_, *v*_g_ represents the group velocity of erbium-doped fiber, there has time point *t_i_*=(*i*−1)Δ*t*, (*i* =1, 2, …, *I* ). It is assumed that the number of particles *n*_2,i,_*_k_*, (*k* =1, 2, …, *K* ) in each segment of erbium-doped fiber is evenly distributed and remains unchanged in ∆*t*. When only forward ASE is considered, the total input light power of each segment are *P*_p,_*_i_*_,_*_k_* + *P*_p,_*_i_*_,_*_k_*, (*P*_p,0,0_=*P*_p_). The forward ASE can be express as:

$P_{\text{ASE,0,0}}^{+}=P_{\text{ASE,}I\text{,}K}^{+}\cdot T\cdot(1-\xi)\cdot(1-cp)$ (S8)

where *T* is the transmission coefficient of the double-fiber-coupled microtubule cavity in the ring cavity, *ξ* is the loss of the ring cavity, and *cp* represents the splitting ratio of the optical coupler. The traveling wave rate equation of numerical ring cavity can be expressed as:

$n_{2,k,i+1}=n_{2,k,i}+\left[W_{\text{p},k,i}(n-n_{2,k,i})-\sum_{m=1}^{M} g_{k,i,m}W_{\text{ASE},k,i,m}-\frac{n_{2,k,i}}{\tau}\right]\times\Delta t$ (S9)

$P_{\text{p},k+1,i+1}=P_{\text{p},k,i}\exp[-\Gamma_{\text{p}}\sigma_{\text{pa}}(n-n_{2,k,i})\Delta z]$ (S10)

$P_{\text{ASE,}k+1,i+1,m}^{+}\text{=}P_{\text{ASE,}k,i,m}^{+}\exp(g_{k,i,m}\Delta z)+2n_{\text{sp},k,i,m}h\upsilon_{m}\exp(g_{k,i,m}\Delta z-1)\Delta\upsilon$ (S12)

$W_{\text{p},k,i}=\frac{\Gamma_{\text{p}}\sigma_{\text{pa}}}{Sh\upsilon_{\text{p}}}\cdot\frac{P_{\text{p},k-1,i}-P_{\text{p},k,i}}{2}$ (S13)

$W_{\text{ASE},k,i,m}=\frac{1}{Sh\upsilon_{\text{p}}}\cdot\frac{P_{\text{ASE,}k-1,i,m}^{+}-P_{\text{ASE,}k,i,m}^{+}}{2}$ (S14)

$g_{k,i,m}=\Gamma_{\text{s}}\sigma_{\text{se}}(\upsilon_{m})\left\{[1+\mu(\upsilon_{m})]n_{2,k,i}-\mu(\upsilon_{m})n\right\}$ (S15)

where *S* is the section area of erbium-doped fiber.


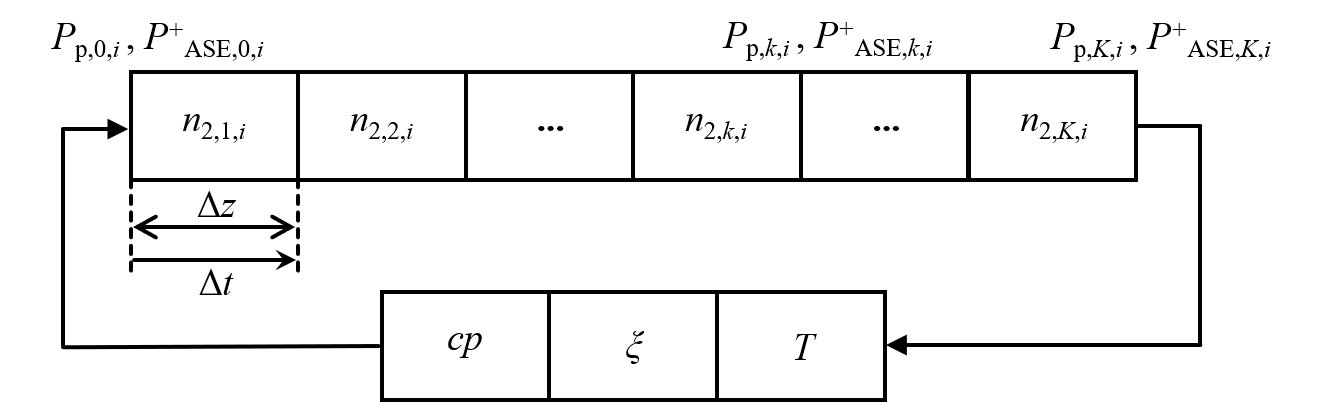


Fig. S5. Schematic diagram of numerical model for fiber ring laser cavity.

Fig. S6 shows the flowchart of numerical solution for fiber ring laser cavity. According to the number of iterations *N*, set the iterative length Δ*z* of the time layer. In each iteration Δ*t*, the input and output powers of each segment of erbium-doped fiber are calculated through sub iterations, and then the metastable particle number of each segment is updated through horizontal sub iterations. The optical power and particle number are obtained through two horizontal sub iterations, which ensures that the input and output powers of each segment are calculated at the same time, that is, the metastable particle number of each erbium-doped fiber segment is not changed. The numerical simulation of sensing laser is realized by MATLAB software. Table S1 gives the relevant parameters of numerical estimation for fiber ring laser cavity. Using the drop reflection of the double-fiber-coupled microtubule cavity as lasing filter (transmission coefficient *T*) of *P*^+^_ASE,0,0_, the simulation results of output sensing laser of fiber ring cavity with drop filter are shown in Fig. S7. Under the frequency selection of microcavity, a single wavelength laser at 1531.163 nm is generated, whose lasing wavelength corresponds to the peak wavelength of drop reflection.


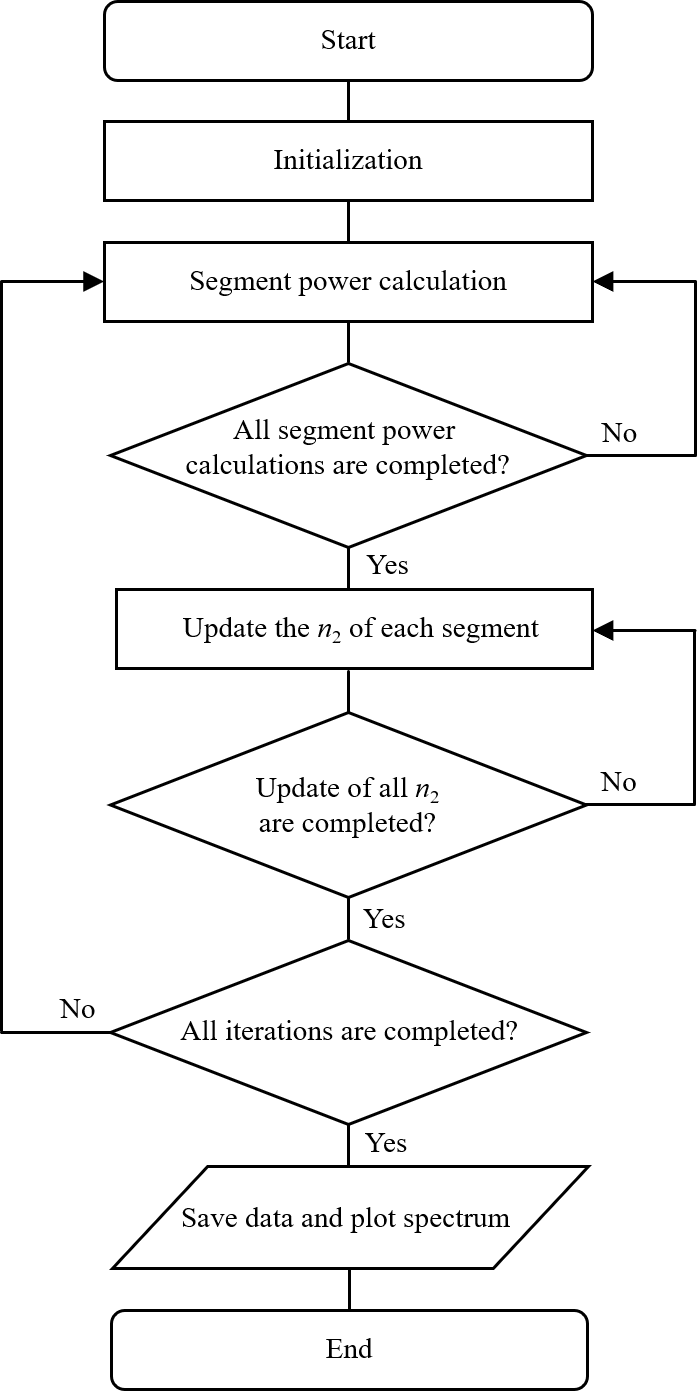


Fig. S6. Flowchart of numerical solution for fiber ring laser cavity.

Table S1. Relevant parameters of numerical estimation for fiber ring laser cavity.

| Parameter | Value | Unit | Parameter | Value | Unit |
| --- | --- | --- | --- | --- | --- |
| *υ*_p_ | 3.06e14 | Hz | Г_p_ | 0.8 | / |
| *P*_p_ | 0.15 | W | Г_s_ | 0.7 | / |
| *L* | 1 | m | *τ* | 0.092 | s |
| *K* | 5 | / | *υ*_p_ | 1.88e14 | Hz |
| *S* | 8.04e-12 | m^2^ | *υ*_p_ | 2.00e14 | Hz |
| *n* | 3.9e25 | m^-3^ | *m* | 2000 | / |
| *v*_g_ | 2.075e8 | m | *ξ* | 0.2 | / |
| σ_pa_ | 3.8e-25 | m^2^ | *cp* | 0.05 | / |


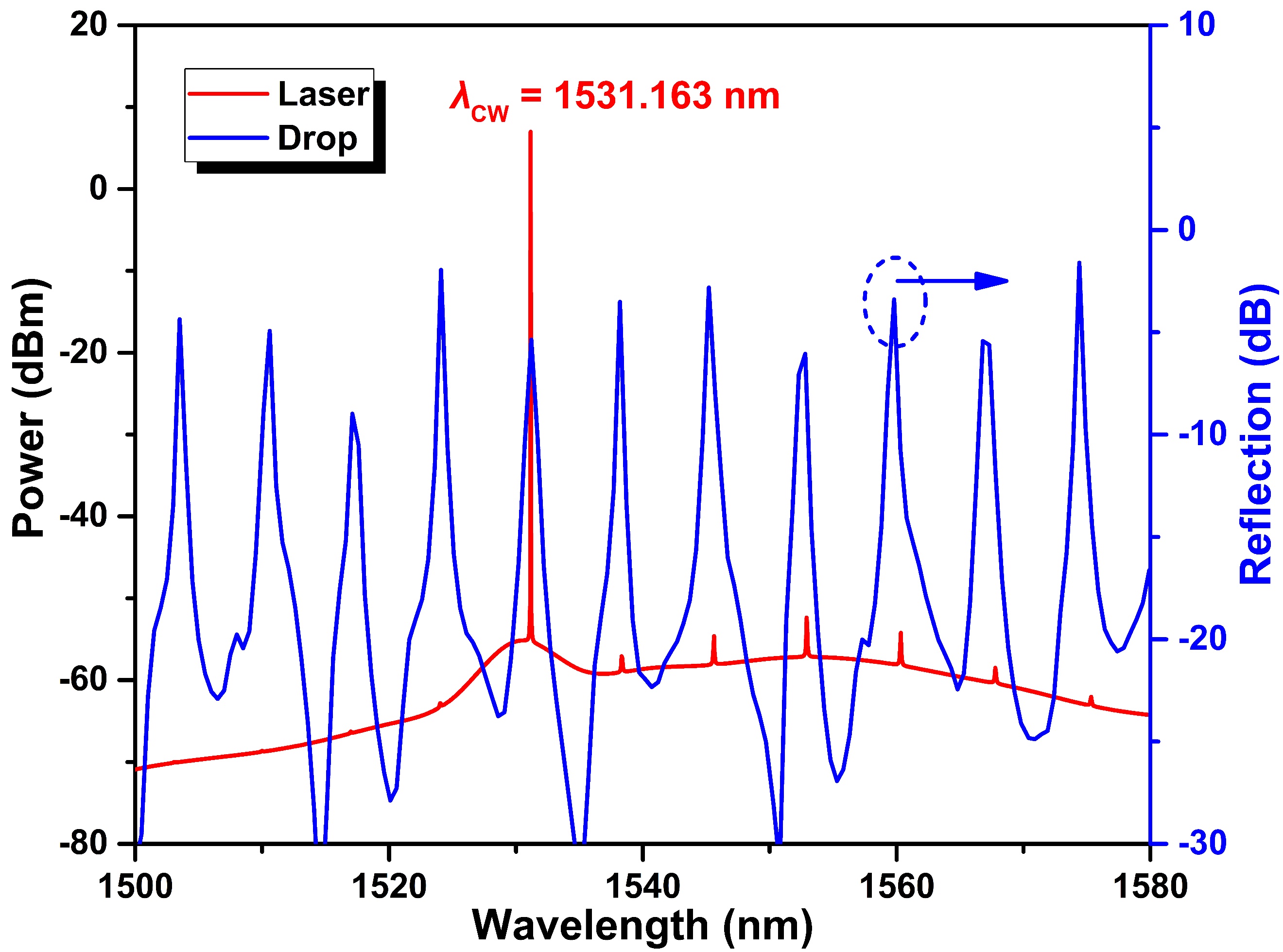


Fig. S7. Simulation results of output laser of fiber ring cavity with drop filter.

*1.3 Numerical estimation of sensing laser wavelength shift*

In our work, the detection of myocardial infarction biomarker is achieved by transducing the biomarker molecule binding into the refractive index change of microcavity. The drop reflection at different refractive indexes was calculated based on the simulation model in Section 1. Fig. S8 shows the simulation results of the through and drop spectra at different refractive indexes, and the response of resonant wavelength to refractive index is given in Fig. S9. The resonant wavelength of simulated through and drop spectra have redshifts with the increase of refractive index in the microtubule, which are consistent with the experimental results. According to the refractive index response of drop, the sensing laser is calculated by setting the simulated drop reflections as the transmission coefficient *T* of *P*^+^_ASE,0,0_ at different refractive indexes, the simulated results are shown in Fig. S10, and the response of lasing wavelength to refractive index is plotted in inset of Fig. S10. Because the sensing laser wavelength is determined by the peak wavelength of drop, the simulated lasing wavelength has similar refractive index response with the resonant wavelength of drop spectrum, and the refractive index sensitivity of drop resonant wavelength and lasing wavelength are 57.949 nm/RIU and 57.836 nm/RIU, respectively. The refractive index sensitivities difference between the experiments and the simulations may be due to the subtle difference of geometric parameters and material properties between the ideal simulation model and the actual fabricated structure.


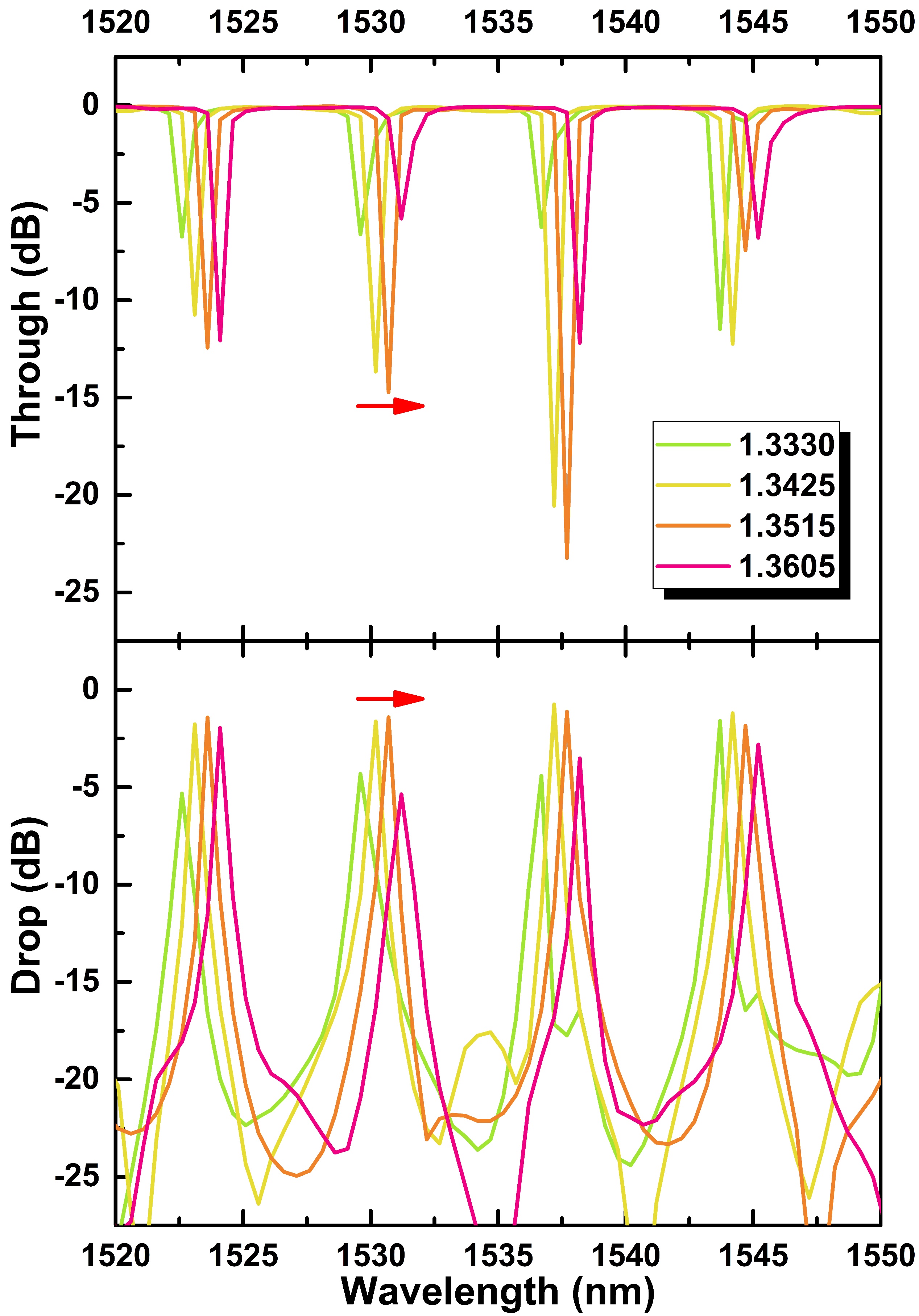


Fig. S8. (a) Simulated results of the through and drop spectra at different refractive indexes.


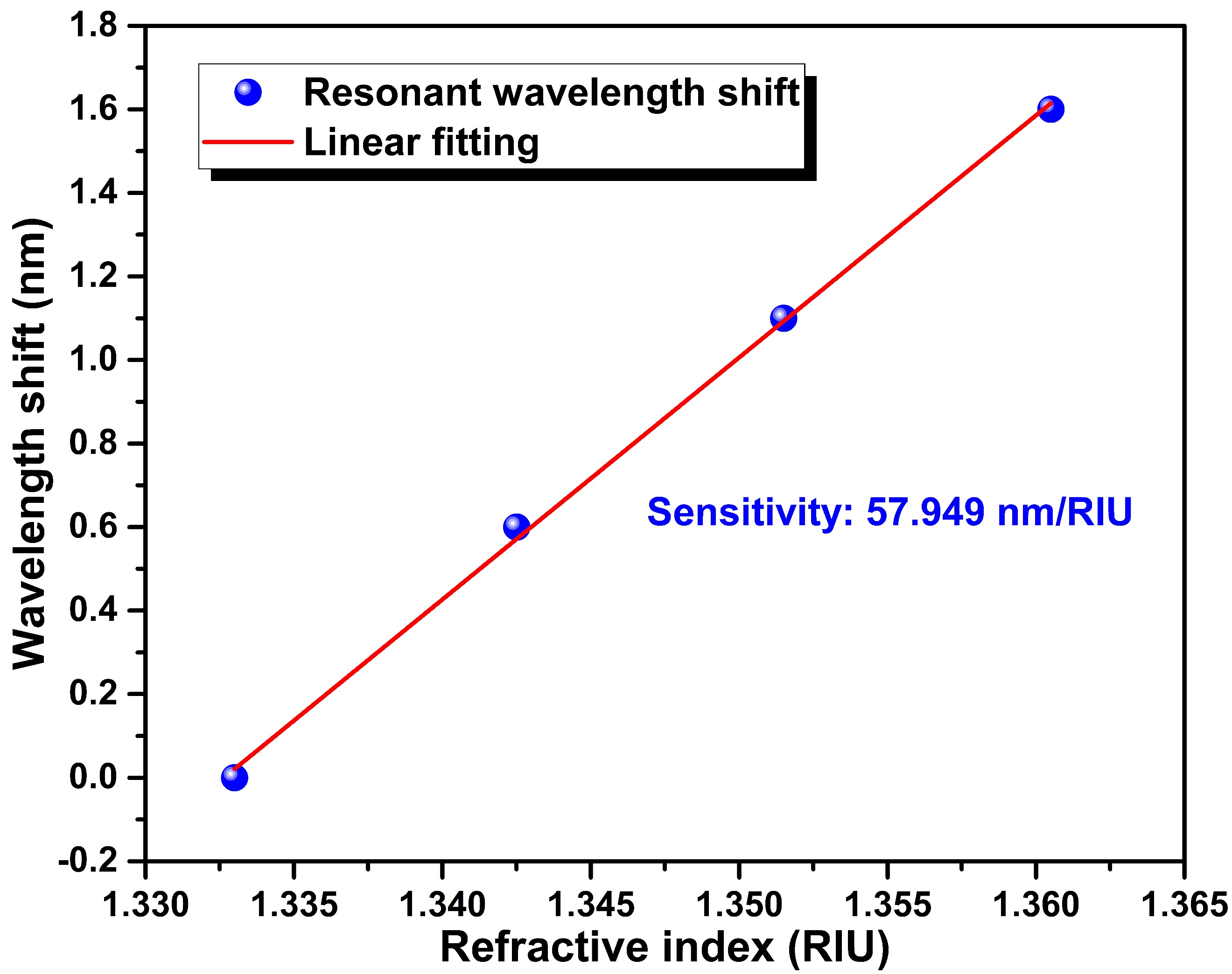


Fig. S9. Response of resonant wavelength to refractive index.


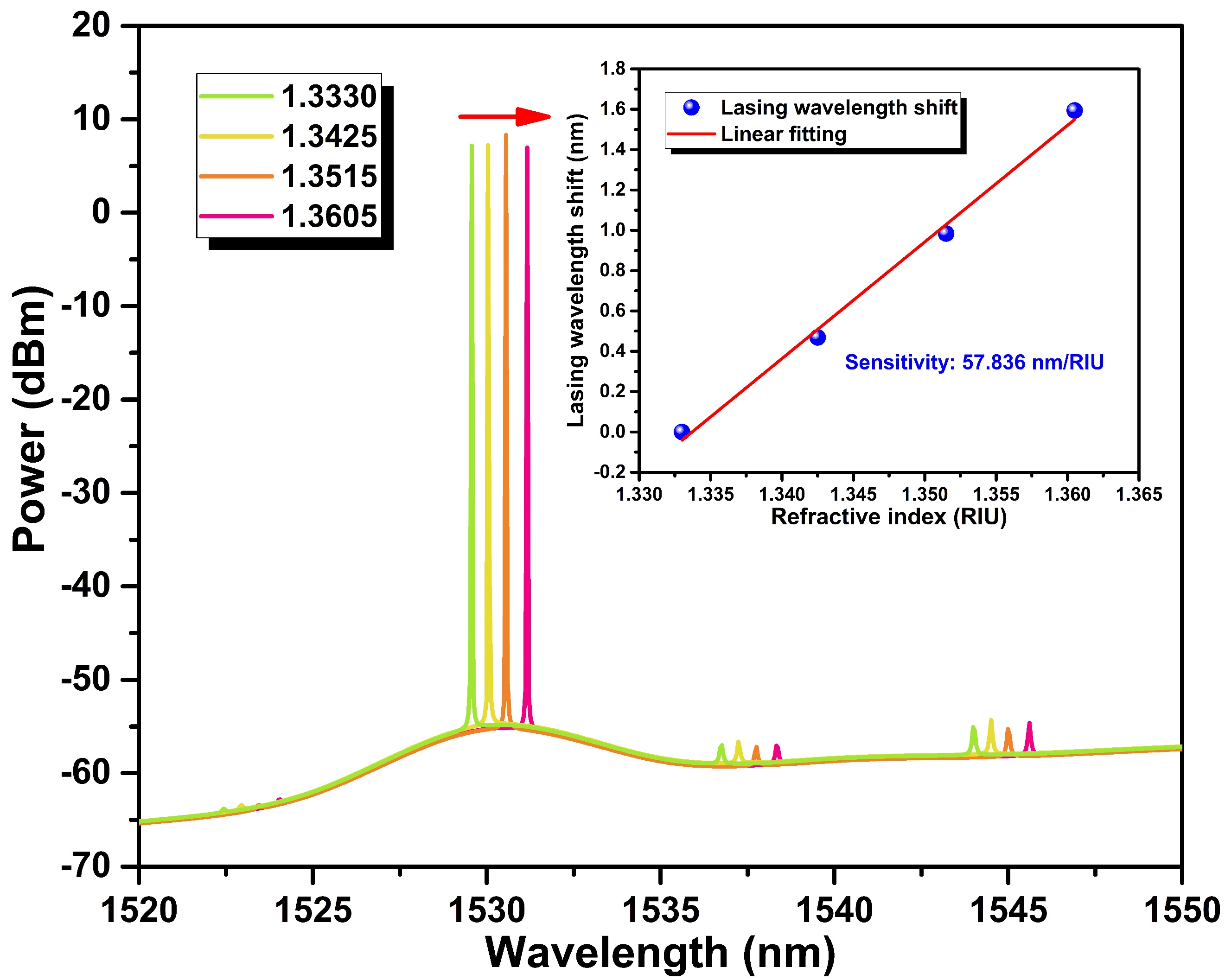


Fig. S10. Simulated results of the sensing laser at different refractive indexes. Inset: Response of lasing wavelength to refractive index.

**Section 3. A summary comparison between the proposed optical fiber biosensor and the recent plasmonic nanostructures and metasurfaces-based optical biosensors.**

**Table S2. A summary comparison between the proposed optical fiber biosensor and the recent plasmonic nanostructures and metasurfaces-based optical biosensors.**

| Scheme description | Detection target | Manufacture method | Optical detection method | Microfluidic channel | Year | Ref. |
| --- | --- | --- | --- | --- | --- | --- |
| Gold nanoantenna arrays loaded with graphene | Immunoglobulin G | Electron-beam deposition and lithography, chemical vapor deposition, e-beam | Fourier Transform Infrared Spectroscopy | Nil | 2019 | [S1] |
| Plastic-templated metasurfaces nanograting | Proteins and HIV viral particles | chemical etch, electron-beam evaporation | Spectrometer with a series of optical components | Additional | 2020 | [S2] |
| Silicon-on-insulator nanorod array | Single-strand oligoDNAs | Negative electron  beam resist, electron beam drawing, deep reactive-ion etching | Fluorescence image by CCD | Additional | 2021 | [S3] |
| Plasmonic nanopillar and nanohole metasurfaces | Tumor markers | Coupled oxygen plasma etching, electron-beam evaporation, reactive oxygen plasma etching | Spectrometer with fiber probes | Additional | 2022 | [S4] |
| Microtubule coupled with double microfibers | Myocardial infarction biomarker | Oxyhydrogen flame tapering | Spectrometer only | Inherent |  | This work |

**References:**

[S1] Z. Li, Y. Zhu, Y. Hao, M. Gao, M. Lu, A. Stein, A. Park, J. Hone, Q. Lin, and N. Yu, "Hybrid metasurface-based mid-infrared biosensor for simultaneous quantification and identification of monolayer protein," ACS Photonics 6, 501−509 (2019).

[S2] R. Ahmed, M. Ozen, M. Karaaslan, C. Prator, C. Thanh, S. Kumar, L. Torres, N. Iyer, S. Munter, S. Southern, T. Henrich, F. Inci, and U. Demirci, "Tunable Fano-resonant metasurfaces on a disposable plastic-template for multimodal and multiplex biosensing," Advanced Materials 32, 1907160 (2020).

[S3] M. Iwanaga, "High-sensitivity high-throughput detection of nucleic acid targets on metasurface fluorescence biosensors," Biosensors 11, 33 (2021).

[S4] F. Li, J. Shen, C. Guan, Y. Xie, Z. Wang, S. Lin, J. Chen, and J. Zhu, "Exploring near-field sensing efficiency of complementary plasmonic metasurfaces for immunodetection of tumor markers," Biosensors and Bioelectronics 203, 114038 (2022).
